# Short‐Chain Fatty Acids Modulate Anti‐ROR1 CAR T‐Cell Function and Exhaustion in an Intestinal Adenocarcinoma‐on‐Chip Model

**DOI:** 10.1002/adhm.202405003

**Published:** 2025-04-18

**Authors:** Valentin D. Wegner, Adrian Feile, Miriam Alb, Michael Hudecek, Philip Hewitt, Alexander S. Mosig

**Affiliations:** ^1^ Institute of Biochemistry II Jena University Hospital 07747 Jena Germany; ^2^ Lehrstuhl für Zelluläre Immuntherapie Medizinische Klinik und Poliklinik II Universitätsklinikum Würzburg 97080 Würzburg Germany; ^3^ Fraunhofer Institut für Zelltherapie und Immunologie (IZI) Außenstelle Würzburg Zelluläre Immuntherapie 97080 Würzburg Germany; ^4^ Chemical and Preclinical Safety Merck Healthcare KGaA 64293 Darmstadt Germany

**Keywords:** CAR T‐cells, intestine‐on‐chip, microbiota, oncology, regulatory T‐cells, tumor‐on‐chip

## Abstract

Chimeric antigen receptor (CAR) T‐cell therapy represents a promising approach for cancer treatment, with receptor tyrosine kinase‐like orphan receptor 1 (ROR1) emerging as a novel target in malignancies. This study investigates how short‐chain fatty acids (SCFAs), key microbiota‐derived metabolites, modulate anti‐ROR1 CAR T‐cell efficacy using a physiologically relevant intestinal adenocarcinoma‐on‐chip model that replicates the human intestinal microenvironment. The findings demonstrate that propionate and butyrate inhibit anti‐ROR1 CAR T‐cell function by reducing infiltration, cytotoxicity, and cytokine release while preserving junctional integrity within the tumor model. Mechanistically, these SCFAs inhibit histone deacetylase activity and promote a phenotype switch toward regulatory T‐cells, as indicated by increased expression of FoxP3 and RORγt. Additionally, propionate and butyrate upregulate PD‐1 and TIM‐3, markers of T‐cell exhaustion and immune tolerance, and induce a dose‐ and time‐dependent reduction in proinflammatory cytokines. In contrast, acetate and pentanoate promote a proinflammatory T helper 17 phenotype. These results highlight the immunomodulatory effects of SCFAs on CAR T‐cell function, emphasizing the need to consider microbiota‐derived metabolites in CAR T‐cell therapies.

## Graphical abstract

Short‐chain fatty acids modulate anti‐ROR1 chimeric antigen receptor (CAR) T‐cell function in an intestinal adenocarcinoma‐on‐chip model. Butyrate and propionate reduce cytotoxicity, cytokine release, and infiltration by inducing a regulatory T‐cell phenotype and T‐cell exhaustion via histone deacetylase inhibition. In contrast, acetate and pentanoate support a proinflammatory Th17 phenotype, underscoring microbiota‐derived metabolite influence on CAR T‐cell efficacy.

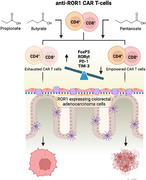

## 1 Introduction

Recent advancements in cancer therapies have significantly enhanced the potential of targeted immunotherapeutic approaches. These include immunotherapies like immune checkpoint inhibitors that block PD‐1/PD‐L1 or CTLA‐4 and chimeric antigen receptor (CAR) T‐cells. These novel therapies have transformed the management of both solid and hematological malignancies, particularly in relapsed/refractory (R/R) malignancy settings.^[^ ^1^, ^2^ ^]^​ Especially CAR T‐cells represent a pivotal development in this area. This therapy using autologous T‐cells, engineered to target specific tumor antigens, has been successfully applied in treating refractory B‐cell hematological malignancies. Despite their promise, the efficacy of these cancer therapies can vary considerably among individuals due to several factors, including on‐target, off‐tumor adverse effects, such as cytokine release syndrome.^[^ ^3^, ^4^ ^]^

Metabolites released by intestinal microorganisms play a crucial role in modulating the host's immune system and response to cancer therapies​​. Recent studies have shown the substantial impact of the gut microbiome on the effectiveness and potential side effects of CAR T‐cell therapy for treating hematologic malignancies.^[^ ^5^ ^]^ Nonclinical and clinical studies have suggested a link between gut microbial diversity and the efficacy of cancer immunotherapy, indicating that gut microbiota modulation could significantly influence responses to CAR T‐cell therapies.^[^ ^6^ ^]^ However, most adoptive T‐cell transfer (ACT) methods involve depleting the immune system through chemotherapy or irradiation,^[^ ^7^ ^]^ which affects the gut microbiota. Studies have shown that these pretreatments of the patient can either amplify or dampen the function of effector T‐cells, depending on the specific microbial communities and their interaction with the immune system.^[^ ^8^ ^]^

Given the intimate crosstalk between the gut microbiota and immune system, microbial metabolites have emerged as key modulators of immune responses, with short‐chain fatty acids (SCFAs) playing a particularly significant role in shaping T‐cell function and homeostasis. SCFAs are natural products of commensal microorganisms, produced by the fermentation of dietary fibers via the gut microbiota. These metabolites, including acetate, propionate, and butyrate, play a significant role in maintaining intestinal homeostasis and modulating the immune system by influencing metabolic status, activating or suppressing signaling pathways, and inducing epigenetic changes.^[^ ^9^ ^]^

Specifically, butyrate promotes the differentiation of T‐regulatory cells (T_regs_), which play a crucial role in maintaining gut homeostasis. T_regs_ exhibit immunosuppressive properties primarily through histone deacetylase (HDAC) inhibition‐related signaling pathways.^[^ ^10^, ^11^ ^]^ This inhibitory effect could potentially limit CAR T‐cell activity.^[^ ^12^ ^]^​ Furthermore, pentanoate and butyrate have also been shown to enhance the antitumor activity of ex vivo‐treated CAR T‐cells.^[^ ^13^ ^]^ This dual effect implies that, while SCFAs can boost CAR T‐cell efficacy under certain conditions, they might also limit efficacy by promoting immune cell exhaustion.^[^ ^14^, ^15^ ^]^ Given this variety of properties of SCFAs, understanding their influence on CAR T‐cell function and therapy outcomes is essential.

This study investigates how the SCFAs, acetate, propionate, butyrate, and pentanoate, modulate CAR T‐cell activity. The experiments were conducted using a human intestinal adenocarcinoma‐on‐chip (IAC) model consisting of an intestine‐on‐chip model mimicking a tumor environment. The model comprises human endothelial cells and macrophages cocultured with colorectal adenocarcinoma CaCo2 cells expressing the receptor tyrosine kinase‐like orphan receptor 1 (ROR1) protein. ROR1 has emerged as a promising target for CAR T‐cell therapy due to its selective expression in various malignancies and minimal presence in normal adult tissues. Initially identified as a fetal antigen involved in embryogenesis, ROR1 is typically absent in most differentiated adult cells. However, aberrant re‐expression of ROR1 has been observed in several epithelial cancers, including colorectal cancer (CRC), where it contributes to tumor progression, epithelial–mesenchymal transition, and metastasis.^[^ ^16^ ^]^

Recent studies have demonstrated that ROR1 is upregulated in intestinal adenocarcinomas and correlates with aggressive tumor characteristics, including enhanced invasiveness and resistance to therapy. Specifically, high ROR1 expression in CRC has been associated with advanced clinical stage, lymph‐node metastasis, and shorter overall survival, indicating its potential as an independent prognostic marker.^[^ ^17^ ^]^

Given the need to improve CAR T‐cell efficacy against solid tumors,^[^ ^18^, ^19^ ^]^ we aim to elucidate the impact of SCFAs on CAR T‐cell cytotoxicity and tumor microenvironment interactions, ultimately contributing to the optimization of CAR T‐cell therapy for intestinal adenocarcinoma.

Our findings revealed that butyrate and propionate decreased the activity of CAR T‐cells by inducing an anti‐inflammatory phenotype in these cells. Additionally, we observed that inhibiting HDAC with the HDAC inhibitor suberoylanilide hydroxamic acid (SAHA) reduced the anticancer activity of CAR T‐cells, mirroring the effects of propionate and butyrate. Further analysis demonstrated that propionate and butyrate inhibited HDAC activity and induced a phenotype switch toward regulatory T‐cells (T_regs_), as reflected by the increased expression of FoxP3 and RORγt. Notably, both metabolites also upregulated the expression of PD‐1 and TIM‐3, key markers of T‐cell exhaustion^[^ ^20^ ^]^ and immune tolerance,^[^ ^21^ ^]^ which was accompanied by a dose‐ and time‐dependent reduction in proinflammatory cytokines. In contrast, acetate and pentanoate did not affect CAR T‐cell activity in the IAC model, and CAR T‐cells treated with these SCFAs exhibited a higher expression of markers associated with a proinflammatory T helper 17 (Th_17_) phenotype. These findings highlight the immunomodulatory effects of SCFAs and their potential to shape CAR T‐cell function within the tumor microenvironment.

## 2 Results

### 2.1 Development and Characterization of the ROR1‐Positive Intestinal Adenocarcinoma‐on‐Chip Model

We leveraged an ROR1‐positive IAC model to study CAR T‐cell activity in a physiologically relevant tumor microenvironment. This model allows studying immune cell interactions under flow conditions to recapitulate dynamic CAR T–cell tumor interaction.

**Figure 1:**
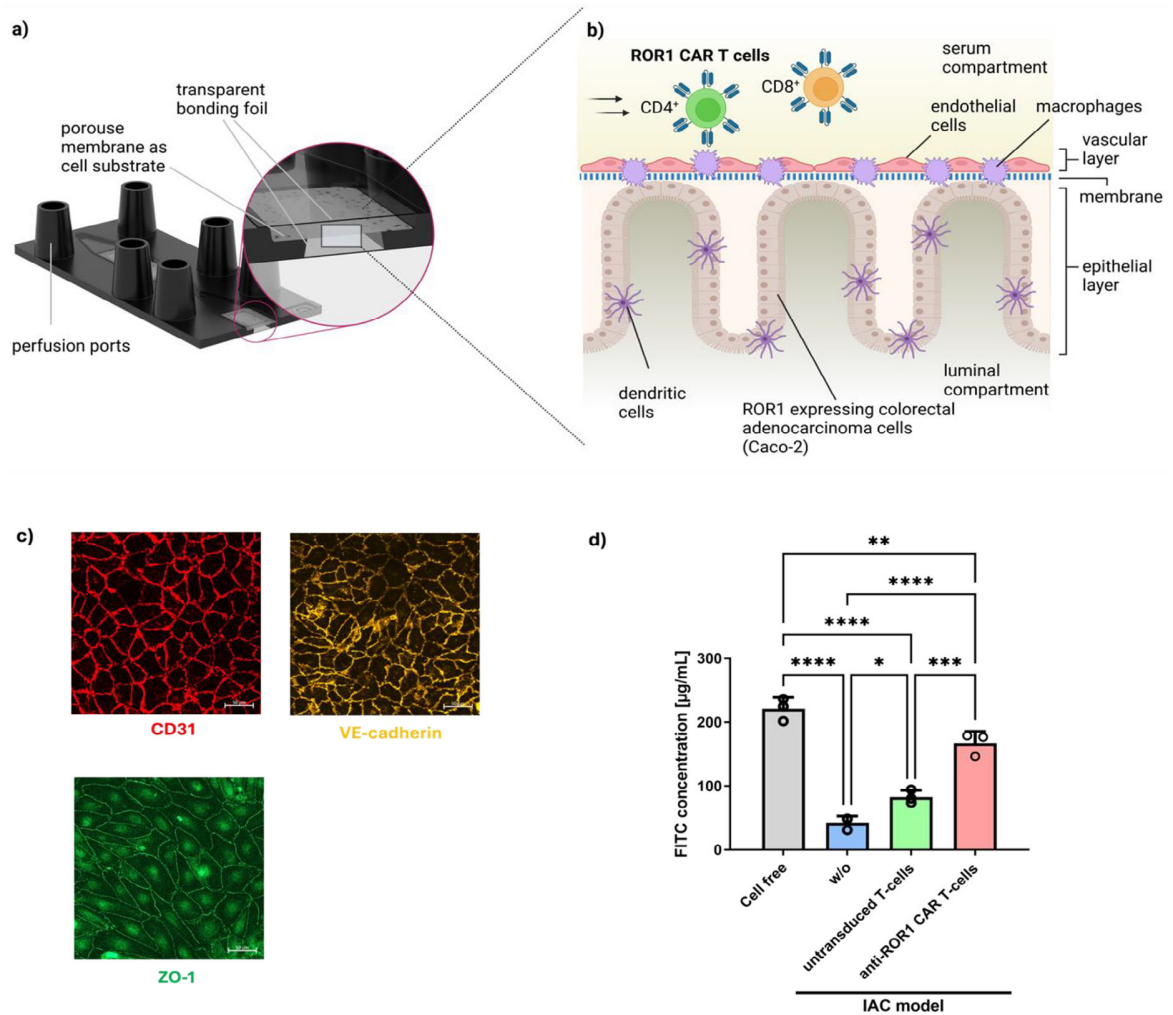
Overview of the IAC model for testing the safety and efficacy of CAR T‐cells. a) Cross‐section of the biochip (created with BioRender.com) and b) schematic overview of the cells present in the IAC model. c) Immunofluorescence staining of the endothelial cell layer forming tight junctions. CD31 is stained in red, VE‐cadherin is stained in yellow, and ZO‐1 is stained in green. Scale bars represent 50 µm. d) Assessment of permeability by FITC‐dextran bead permeation assay of cell‐free microfluidic chips and in the IAC model without T‐cells (w/o), with untransduced T‐cells or anti‐ROR CAR T‐cells.

The IAC model consists of Caco‐2 cells, a human epithelial colorectal adenocarcinoma cell line that forms a 3D epithelial tissue. Previous work has demonstrated that this model provides a suitable platform for studying microbiota–host interactions.^[^ ^22^ ^]^ Under flow conditions, this tissue exhibits crypt‐ and villus‐like structures in the biochip. Macrophages integrated into the epithelial cell layer mimic mucosal immunity and improve barrier formation and epithelial polarization by actively sensing microbial‐associated molecular patterns, such as lipopolysaccharides (LPS). The epithelial cell layer was cultured on a membrane as a cell substrate. The opposite side of the membrane was lined with endothelial cells that resemble a vascularization of the IAC model, forming a tight barrier and facilitating the circulation of CAR T‐cells to simulate the in vivo‐like dynamic interaction of lymphocytes with tumor cells (**Figure** **1**). Endothelial cells were shown to express junctional proteins CD31, VE‐cadherin, and ZO‐1 and to maintain a biological barrier functionality w/o T‐cells and in the presence of untransduced T‐cells from healthy donors. The barrier functionality was significantly diminished upon perfusion with anti‐ROR1 CAR T‐cells (Figure 1c,d).

The IAC model was designed to test the modulation of CAR T‐cell activity by microbiota‐derived SCFAs. As proof of concept, the CAR T‐cells used in this study are directed against ROR1, a cancer‐associated receptor typically expressed in intestinal adenocarcinomas. Therefore, we initially confirmed the expression of ROR1 by Caco‐2 and human umbilical vein endothelial cell (HUVEC) to validate these cells as suitable targets for subsequent studies on CAR T‐cell activity (Figure S1, Supporting Information).

**Figure 2:**
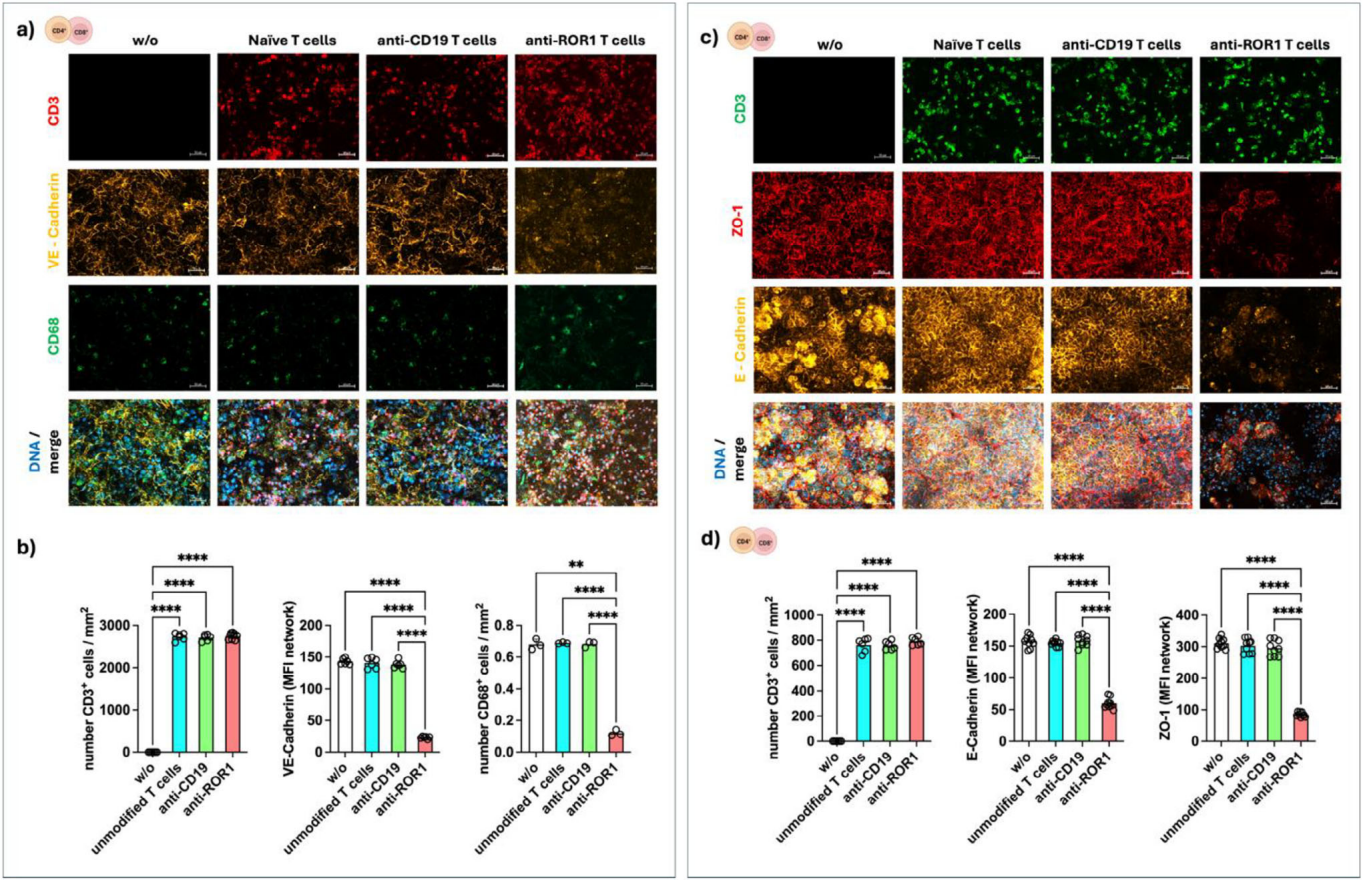
Cytotoxic effects of anti‐ROR1 CAR T‐cells in IAC model. a,b) The vascular side of the IAC model was stained for CD3 (T‐cells, red), VE‐cadherin (endothelial junctions, yellow), and CD68 (macrophages, green). c,d) The epithelial layer of the IAC models was stained for CD3 (T‐cells, green), E‐cadherin (epithelial junctions, yellow), and ZO‐1 (tight junctions, red). Merged channels show nuclei in addition (DAPI, blue). Quantification of biomarkers b,d) confirms that anti‐ROR1 CAR T‐cells, but not anti‐CD19 CAR T‐cells or untransduced T‐cells, induced a significant reduction in VE‐cadherin, E‐cadherin, and ZO‐1 expression. Scale bar represents 50 µm. Statistical significance was determined using a one‐way ANOVA with Tukey's multiple comparison test. Bars represent mean ± SD of 3 independent experiments (*n* = 3) with three data points per replicate. ****p < 0.0001.

CAR T‐cells were circulated on the vascular side of the IAC, with the endothelial lining mimicking the barrier of the blood vessels. We quantified the adhesion of CAR T‐cells at the vascular side and their infiltration into the epithelial cell layer (**Figure** **2**). Cytotoxic effects of CAR T‐cells against target cells were evaluated and quantified by assessing the loss of the cell‐type‐specific apical junctional network protein VE‐cadherin on endothelial cells and E‐cadherin and ZO‐1 on epithelial cells. Furthermore, the loss of the macrophage maturation marker CD68 was assessed. We used untransduced control T‐cells as a control to differentiate the potential baseline effects of allogenic lymphocyte reactions. The potential off‐target effects of engineered CAR T‐cells were investigated using anti‐CD19 CAR T‐cells from the same donor. All tested lymphocytes had a similar T‐cell adhesion and infiltration rate into the enterocyte layer. A cytotoxic effect, which was reflected by the loss of VE‐cadherin, E‐cadherin, and ZO‐1 expression, compared to untransduced control T‐cells and anti‐CD19 CAR T‐cells, was only observed for anti‐ROR1 CAR T‐cells. These results confirmed the CAR T‐cell specificity in the IAC model. An associated depletion of CD68‐positive macrophages upon the addition of anti‐ROR1 CAR T‐cells showed that CAR T‐cell‐mediated cytotoxicity was extended to cells involved in the immune component of the tumor model microenvironment (Figure 2).

### 2.2 Modulation of Tumor Proliferation and Inflammatory Response by Short‐Chain Fatty Acids

**Figure 3:**
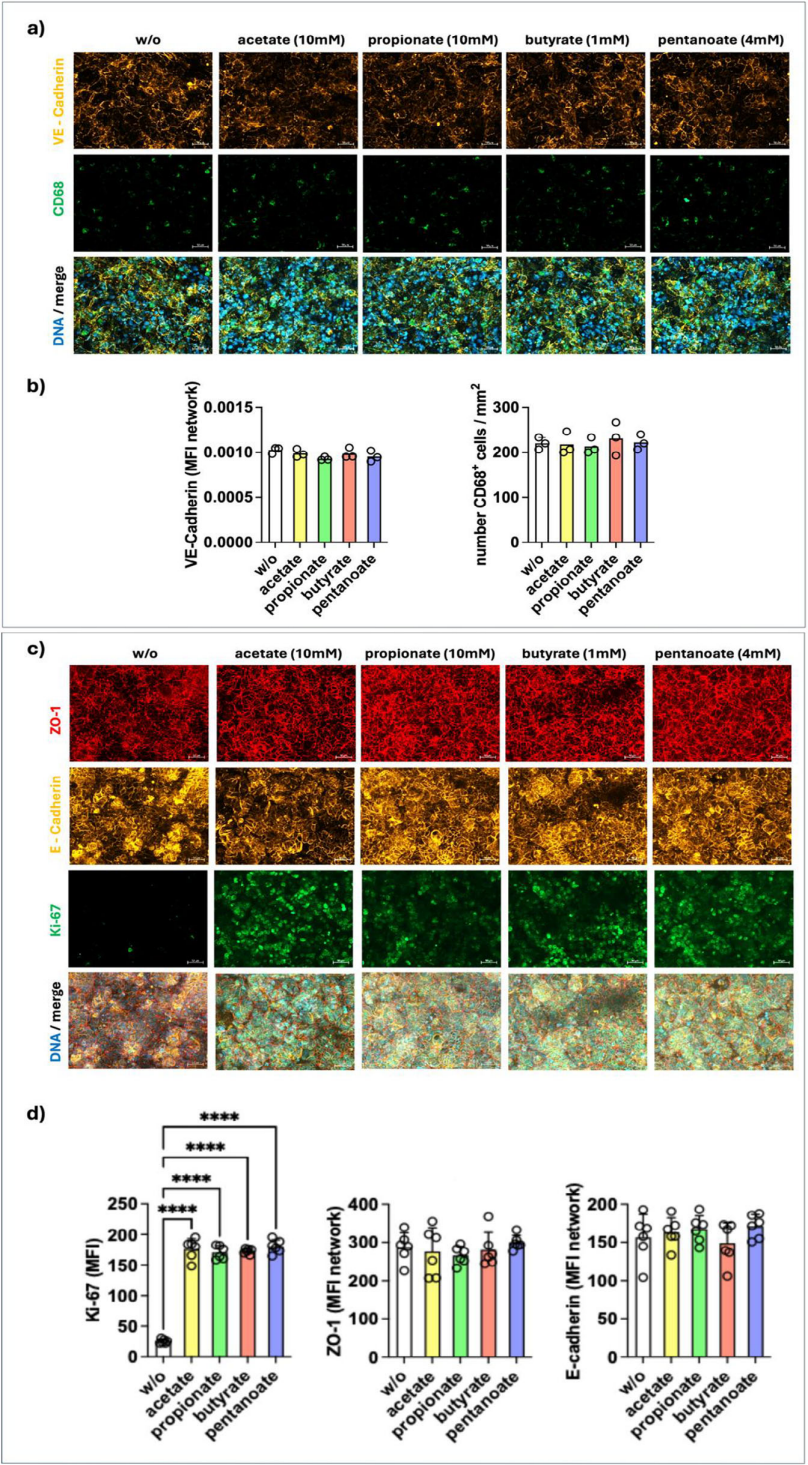
SCFA treatment did not alter junctional protein expression or macrophage counts in the IAC model. a) Vascular layer: VE‐cadherin (yellow) marks endothelial cell junctions, and CD68 (green) identifies macrophages. c) Epithelial layer: ZO‐1 (red) stains tight junctions, E‐cadherin (yellow) marks adheres junctions, and Ki‐67 (green) indicates proliferating epithelial cells. b,d) Quantification of biomarkers. Data are presented as mean ± SD. *n* = 3 independent experiments. Scale bars represent 50 µm. Statistical significance was assessed using one‐way ANOVA with Tukey's multiple comparison test. ****p < 0.0001.

**Figure 4:**
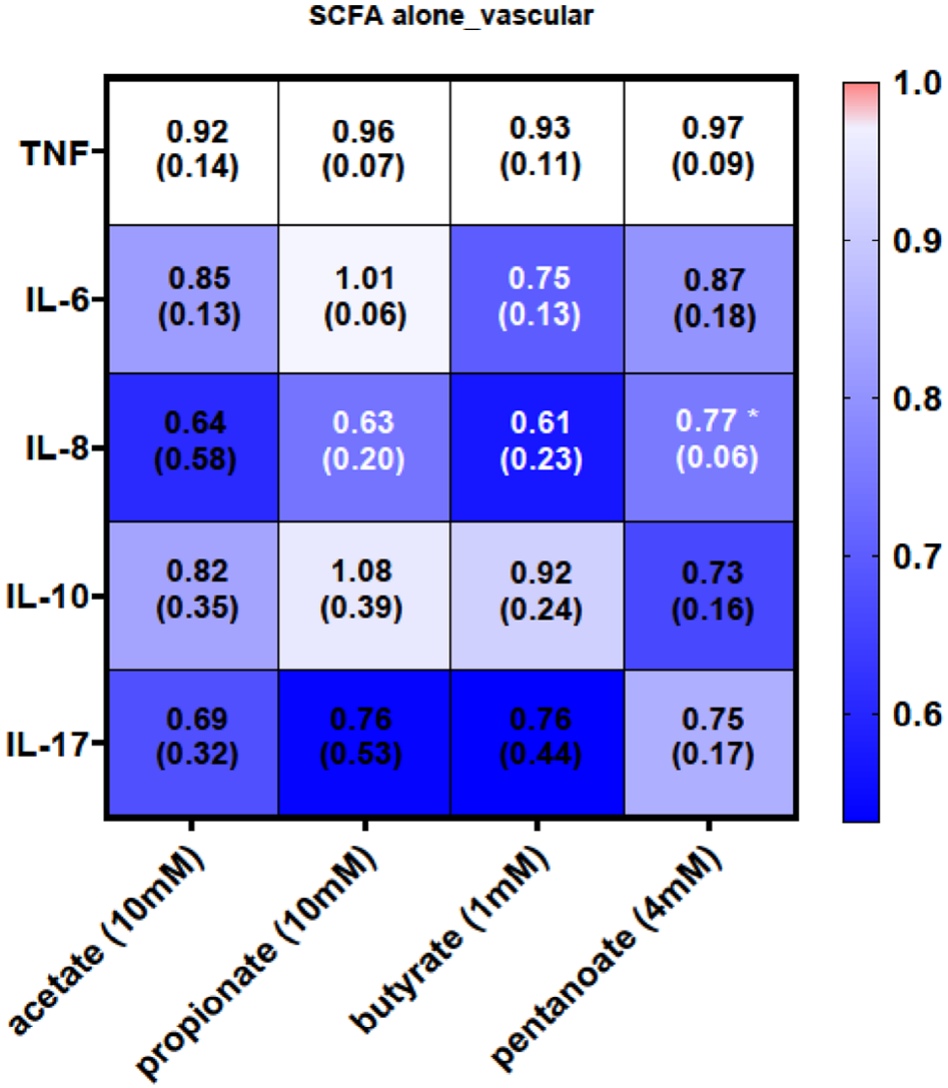
Cytokine profiles of the SCFA‐treated IAC model. SCFAs induce a trend for decreased expression of IL‐6, IL‐8, and IL‐10. Values were normalized against an untreated control and statistical significance was determined by using a one‐way ANOVA with Tukey's multiple comparison test. Values represent mean ± SD of 3 independent experiments (*n* = 3). *p < 0.05.

We investigated the effects of SCFAs, including acetate, propionate, butyrate, and pentanoate, on the IAC model. These metabolites are abundant in the gut lumen and play a significant role in modulating epithelial cell behavior and immune responses.^[^ ^9^ ^]^ SCFA treatment at physiological concentrations typically observed in the gut lumen (luminal level) had no effects on the expression of apical junctional complex proteins (AJCPs) VE‐cadherin, E‐cadherin, and ZO‐1 or the number of CD68‐positive macrophages in the IAC model (**Figure** **3**). However, as seen by the elevated Ki67 staining, SCFA treatment significantly increased the proliferation of enterocytes (Figure 3). This aligns with the in vivo situation, where SCFAs represent a significant energy source for enterocytes.^[^ ^23^, ^24^ ^]^ In addition, we confirmed the anti‐inflammatory effects of SCFAs in our IAC model by demonstrating a trend toward decreased secretion of the proinflammatory cytokines IL‐6 and IL‐8 (**Figure** **4**).

Following the IAC characterization under SCFA treatment, we studied the effect of SCFAs on CD4^+^ and CD8^+^, as well as on a 1:1 mixture of CD4^+^ and CD8^+^ CAR T‐cell subtypes, to gain insights into how microbiota‐derived metabolic products modulate T‐cell function in this IAC model. However, given the established modulatory effects of SCFAs on adenocarcinoma cells, we designed follow‐up studies to analyze the impact of SCFAs on CAR T‐cells without any potential bias of the effects on the IAC model. Therefore, CAR T‐cells were preincubated with SCFAs for 24 or 72 h outside of the IAC model before perfusion to ensure that any changes in CAR T‐cell activity were due to SCFA exposure and not secondary effects from the IAC model or its microenvironment.

To mimic in vivo relevant conditions, we employed two different concentration ranges of SCFA present after gut microbial fermentation of dietary fibers: serum‐level concentrations, which are typically found in the bloodstream, and luminal‐level concentrations, which represent the higher SCFA levels present in the gut lumen. To mimic serum‐level SCFAs, we used 500 µm acetate, 50 µm propionate, 10 µm butyrate, and 10 µm pentanoate for treatment of CAR T‐cells (serum level).^[^ ^25^, ^26^ ^]^ These levels reflect the lower systemic concentrations of SCFAs that may circulate in the blood following gut microbial fermentation of dietary fibers. In contrast, the luminal‐level SCFAs concentrations chosen were substantially higher, with 10 mm acetate, 10 mm propionate, 1 mm butyrate, and 4 mm pentanoate used to replicate SCFA levels typically found in the gastrointestinal tract (luminal level) affecting immune cells in the gut‐associated lymphoid tissue.^[^ ^13^, ^27^, ^28^ ^]^

**Figure 5:**
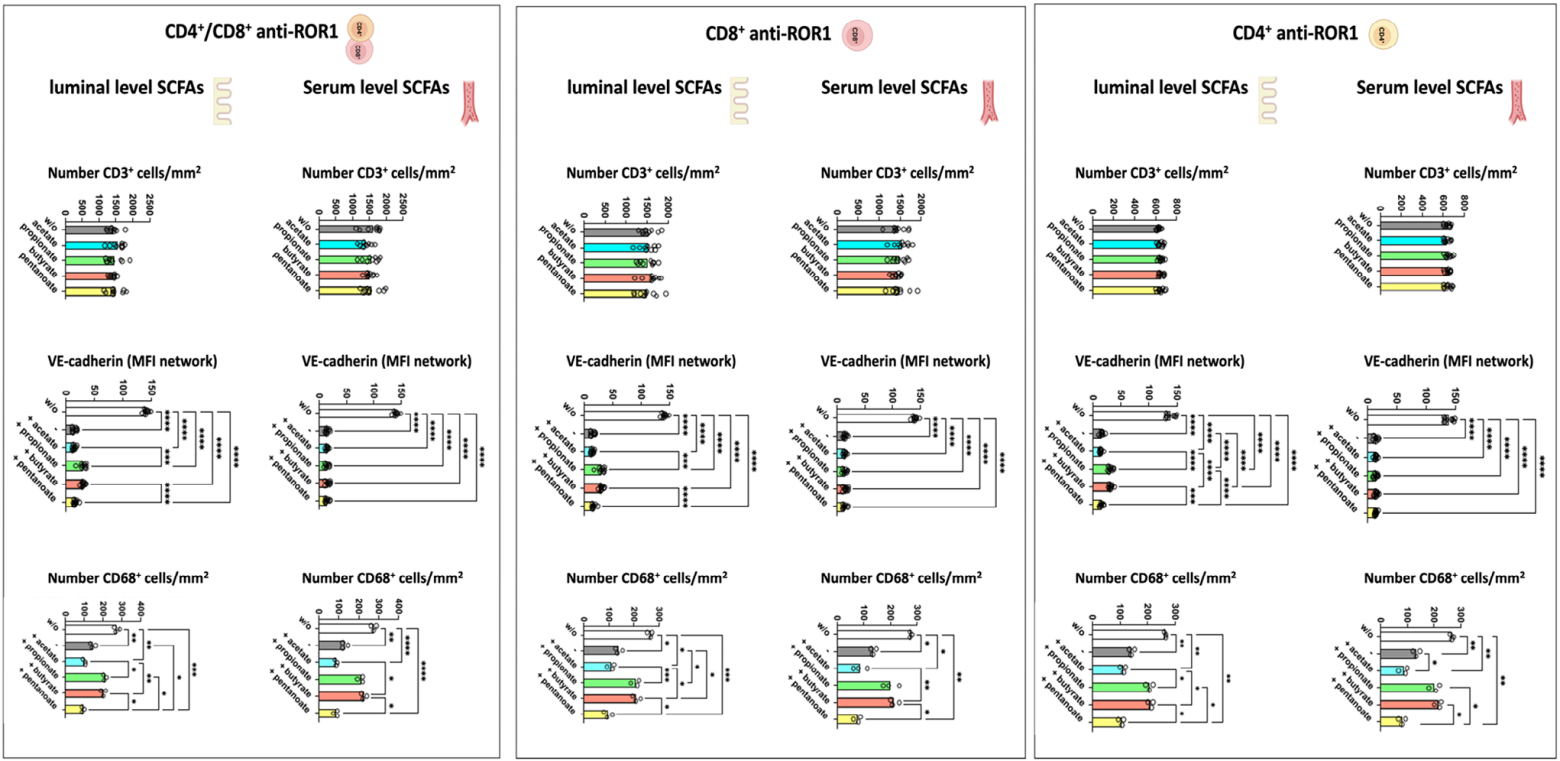
SCFA treatment reduces the cytotoxic effects of anti‐ROR1 CAR T‐cells on the vascular layer in the IAC model. Anti‐ROR1 CAR T‐cells were pretreated with serum‐ or luminal‐level concentrations of acetate, propionate, butyrate, or pentanoate for 24 h before circulation within the IAC model. Quantification of immunofluorescence at the vascular cell layer of T‐cell adhesion (CD3^+^ cells), VE‐cadherin expression, and number of macrophages (CD68^+^ cells) from data shown in Figures S3–S8 of the Supporting Information. Statistical significance was determined using a one‐way ANOVA with Tukey's multiple comparison test. Bars represent mean ± SD of 3 independent experiments (*n* = 3) with three data points per replicate. *p < 0.05, **p < 0.01, ***p < 0.001, ****p < 0.0001.

**Figure 6:**
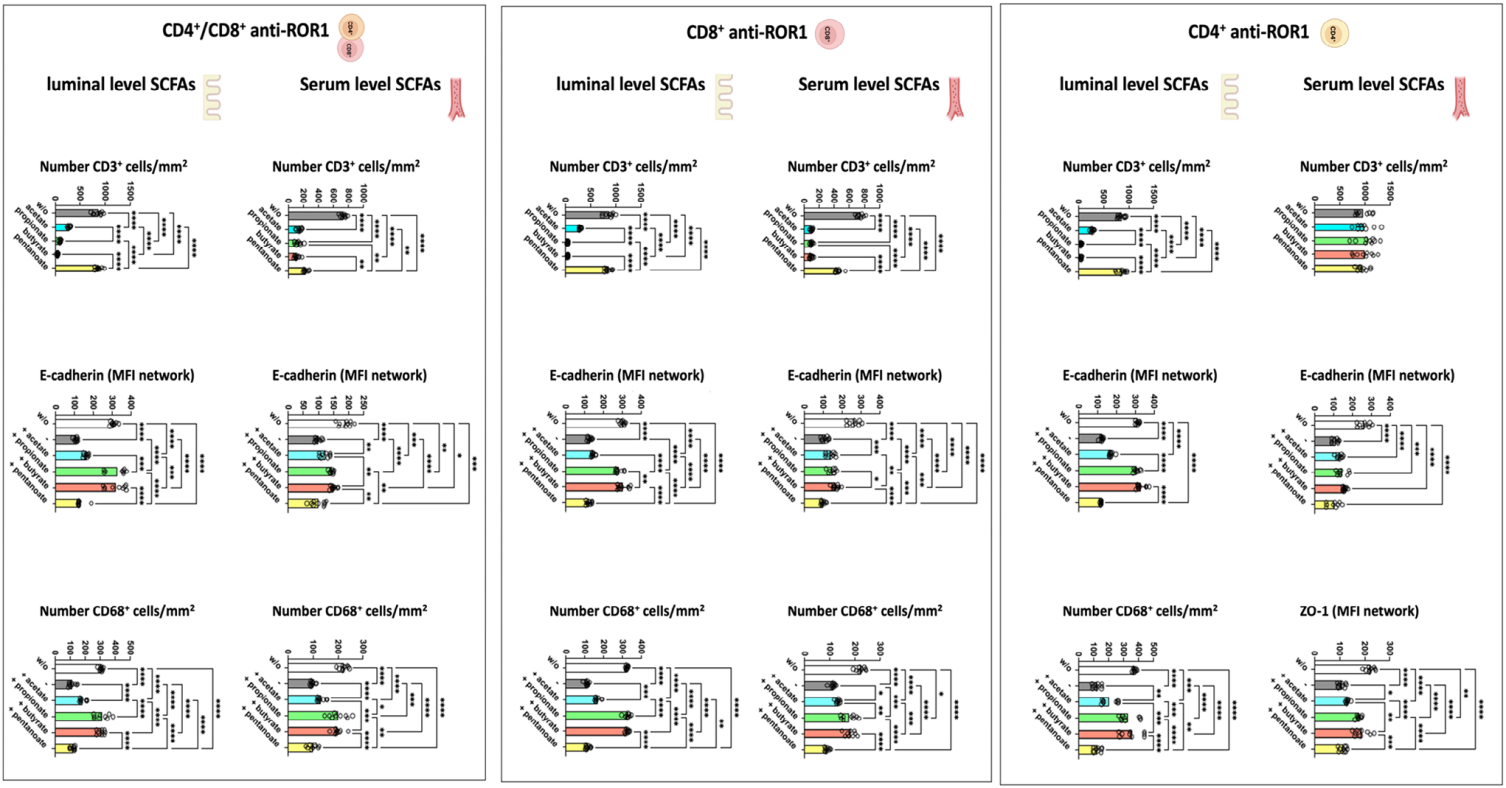
SCFA treatment reduces the cytotoxic effects of anti‐ROR1 CAR T‐cells on the vascular layer in the IAC model. Anti‐ROR1 CAR T‐cells were pretreated with serum‐ or luminal‐level concentrations of acetate, propionate, butyrate, or pentanoate for 24 h before circulation within the IAC model. Quantification of immunofluorescence at the epithelial cell layer of T‐cell infiltration (CD3^+^ cells), E‐cadherin, and ZO‐1 expression from data shown in Figures S3–S8 of the Supporting Information. Statistical significance was determined using a one‐way ANOVA with Tukey's multiple comparison test. Bars represent mean ± SD of 3 independent experiments (*n* = 3) with three data points per replicate. *p < 0.05, **p < 0.01, ***p < 0.001, ****p < 0.0001.

Preincubation of anti‐ROR1 CAR T‐cells for 24 h with luminal level concentrations of the SCFAs propionate and butyrate, but not acetate or pentanoate, partially inhibited the ability to target and eliminate ROR1‐expressing endothelial cells effectively. In addition, when CAR T‐cells were preincubated with propionate or butyrate at both serum and luminal concentrations, their capacity to attack ROR1‐expressing enterocytes was significantly reduced. In contrast, preincubation with acetate and pentanoate had no observable impact on CAR T‐cell cytotoxic activity. The diminished antitumor efficacy of propionate‐ or butyrate‐treated CAR T‐cells was associated with a significantly reduced infiltration rate of CAR T‐cells into the enterocyte layer of the IAC model (**Figures** **5** and **6**; Figures S3–S8, Supporting Information).

**Figure 7:**
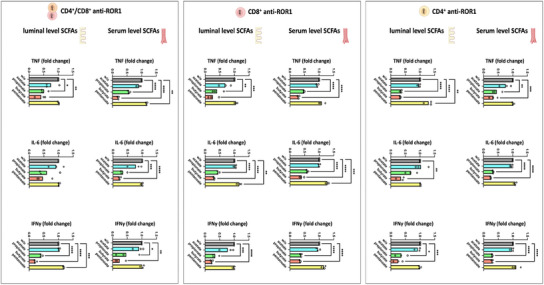
Cytokine release in the IAC model after perfusion with 24‐h SCFA‐pretreated anti‐ROR1 CAR T‐cells. Anti‐ROR1 CAR T‐cells were pretreated with serum‐ or luminal‐level concentrations of acetate, propionate, butyrate, or pentanoate for 24 h before circulation within the IAC model. Statistical significance was determined using a one‐way ANOVA with Tukey's multiple comparison test. Bars represent mean ± SD of 3 independent experiments (*n* = 3) with three data points per replicate. *p < 0.05, **p < 0.01, ***p < 0.001, ****p < 0.0001.

The findings suggest that propionate and butyrate significantly impair the infiltration and function of CAR T‐cells, thereby potentially limiting their therapeutic efficacy in targeting tumor cells. Recent findings have highlighted the immunosuppressive effects of propionate and butyrate on immune cell function, which aligns with our observations.^[^ ^29^, ^30^, ^31^ ^]^ Inhibition of CAR T‐cell infiltration into the ROR1‐expressing enterocyte layer of the IAC model occurred after 24 h of separate preincubation of CAR T‐cells with propionate or butyrate. This was accompanied by a suppressed release of proinflammatory cytokines, including TNF, IL‐6, and IFN‐γ (**Figure** **7**). This suppression was evident in both CD4^+^ and CD8^+^ CAR T‐cells, as well as in mixed CD4^+^/CD8^+^ CAR T‐cell cultures, indicating a broad inhibitory effect of propionate and butyrate on CAR T‐cell‐mediated immune responses within the IAC model. The inhibition of cytokine release and tumor infiltration likely contributes to the reduced antitumor activity of CAR T‐cells.

The SCFA exposure time was extended further to explore the impact of the pretreatment time on CAR T‐cell function. We focused specifically on butyrate, where we observed the most prominent inhibitory effects, and pentanoate, where we found no significant effects within 24 h of CAR T‐cells preincubation. We have tested serum levels and luminal levels of the individual SCFAs on antitumor CAR T‐cell activity.

**Figure 8:**
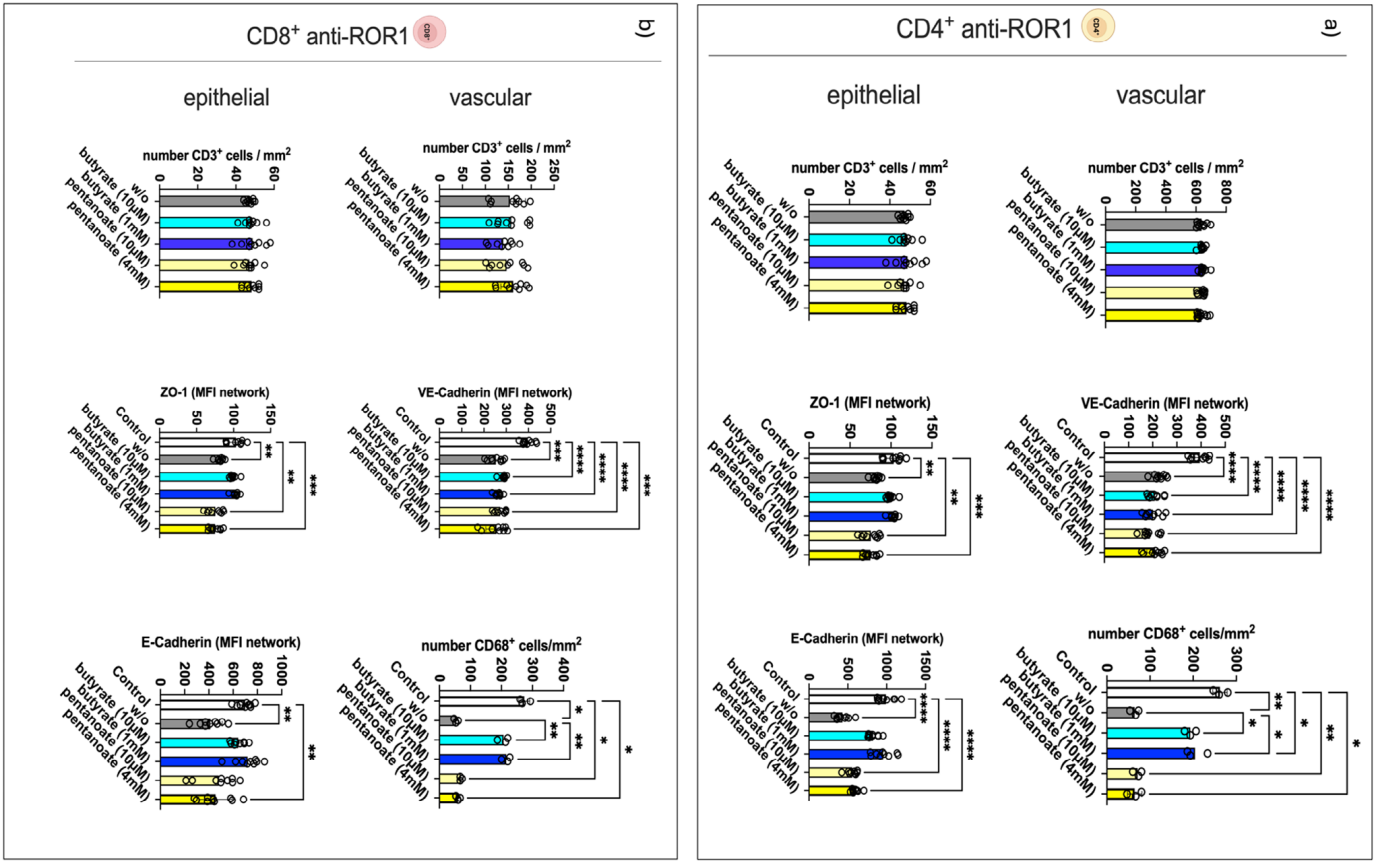
Modulation of CD4^+^ and CD8^+^ anti‐ROR1 CAR T‐cell activity against the IAC model upon pretreatment for 72 h with serum and luminal levels of butyrate and pentanoate. Anti‐ROR1 CAR T‐cells were pretreated with serum‐ or luminal‐level concentrations of butyrate, or pentanoate for 72 h before circulation within the IAC model. Quantification of immunofluorescent stained images. Statistical significance was determined by using a one‐way ANOVA with Tukey's multiple comparison test. Bars represent mean ± SD of 3 independent experiments (*n* = 3) with three data points per replicate. *p < 0.05, **p < 0.01, ***p < 0.001, ****p < 0.0001.

Similar to 24 h preincubation, butyrate inhibited CAR T‐cell activity 72 h after CAR T‐cell pretreatment. While the extension of butyrate pretreatment of CAR T‐cells did not alter CD68‐positive macrophage counts, VE‐cadherin expression of endothelial cells declined upon CAR T‐cell perfusion. Notably, although prolonged butyrate pretreatment did not reduce CAR T‐cell infiltration rates into the enterocyte layer, it effectively inhibited tumor cell targeting, reflected by the preservation of E‐cadherin and ZO‐1 expression, which was similar to the control IAC model without CAR T‐cell perfusion (**Figure** **8**; Figures S10 and S11, Supporting Information). In contrast, pentanoate continued to show no effect on either CAR T‐cell infiltration or antitumor activity at both tested concentrations.

**Figure 9:**
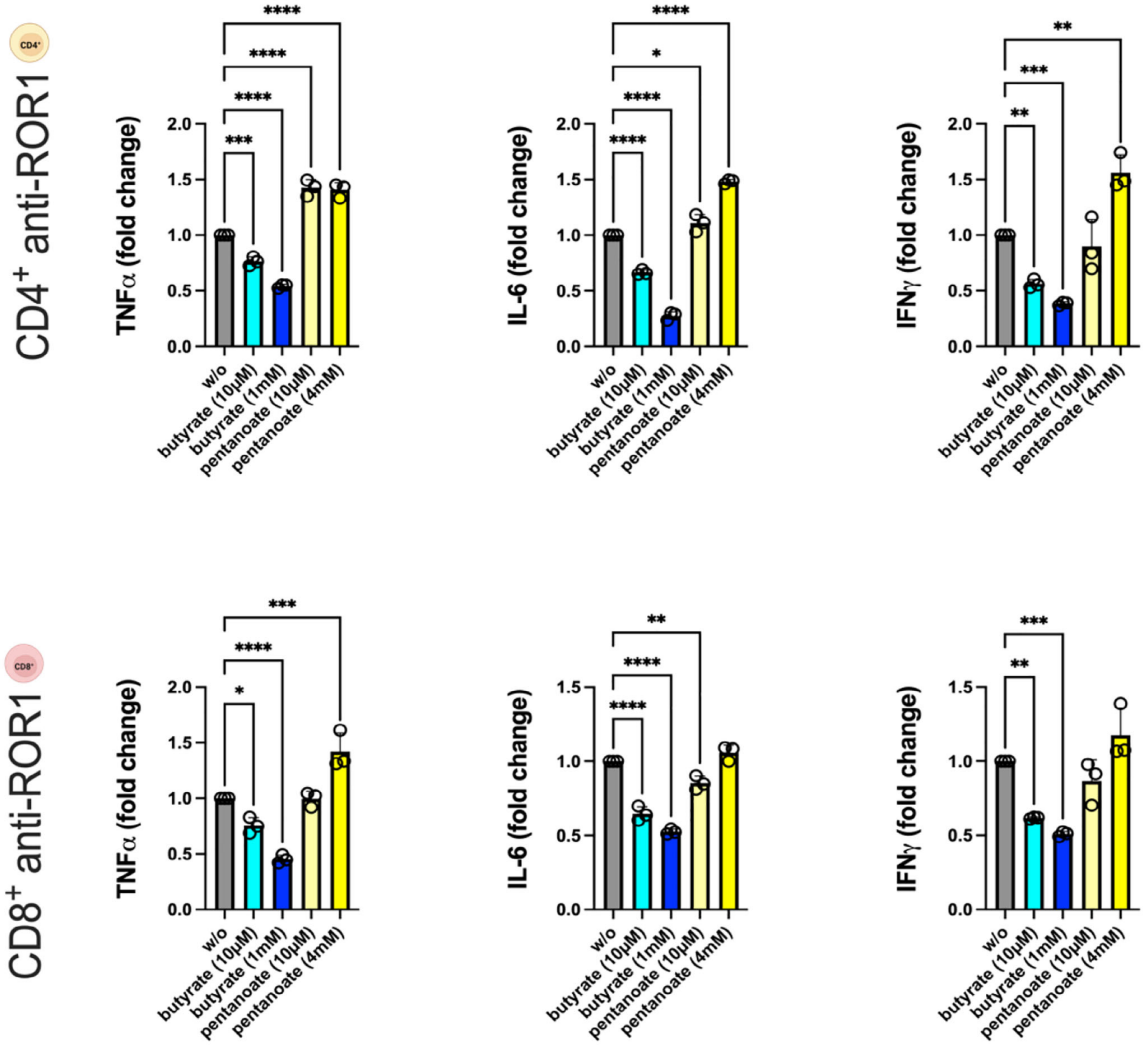
Cytokine release of the IAC model perfused with a combination of CD4^+^ and CD8^+^ anti‐ROR1 CAR T‐cells after 72 h of preincubation with serum and luminal levels of butyrate and pentanoate. Anti‐ROR1 CAR T‐cells were pretreated with serum‐ or luminal‐level concentrations of butyrate, or pentanoate for 72 h before circulation within the IAC model. Statistical significance was determined using a one‐way ANOVA with Tukey's multiple comparison test. Bars represent mean ± SD of 3 independent experiments (*n* = 3) with three data points per replicate. *p < 0.05, **p < 0.01, ***p < 0.001, ****p < 0.0001.

The prolonged preincubation with butyrate significantly reduced the secretion of TNF, IL‐6, and IFN‐γ in a dose‐dependent manner for both pretreated CD4^+^ and CD8^+^ anti‐ROR1 CAR T‐cells compared to untreated anti‐ROR1 CAR T‐cells. In an interesting divergence, pentanoate preincubation, which had shown no impact on CAR T‐cell activity in previous short‐term stimulation assays, led to an increase in proinflammatory cytokine release after 72 h of preincubation for all three cytokines in CD4^+^ CAR T‐cell perfused IACs and for TNF after perfusion with preincubated CD8^+^ CAR T‐cells (**Figure** **9**). These findings demonstrate that butyrate has a prolonged and broad immunosuppressive effect on CAR T‐cell function, while pentanoate could induce a proinflammatory response after prolonged preincubation.

To investigate the underlying mechanisms contributing to the observed effects of SCFAs on lymphocyte activity, we examined potential changes in CAR T‐cell immunometabolism. Despite the known influence of SCFAs on metabolic pathways, we observed no significant alterations in acetyl‐CoA formation or the cellular ADP/ATP ratio of CAR T‐cells incubated for 24 h or 72 h with SCFAs, at both serum and luminal SCFA concentrations (Figure S12, Supporting Information).

These findings suggest that SCFA exposure does not affect the metabolic function of CAR T‐cells at tested concentrations and treatment times, indicating that SCFAs may not mediate immunosuppressive effects through direct modulation of cellular energy metabolism.

Further analysis of the CAR T‐cell phenotype of CD4^+^ and CD8^+^ CAR T‐cells showed that CD25, the interleukin‐2 receptor α‐chain and a key marker associated with T‐cell activation, which is critical for T‐cell proliferation and survival^[^ ^32^ ^]^ was not altered by SCFA treatment, regardless of SCFA concentration (serum‐level or luminal‐level) or exposure duration (24 or 72 h) (Figure S13, Supporting Information).

**Figure 10:**
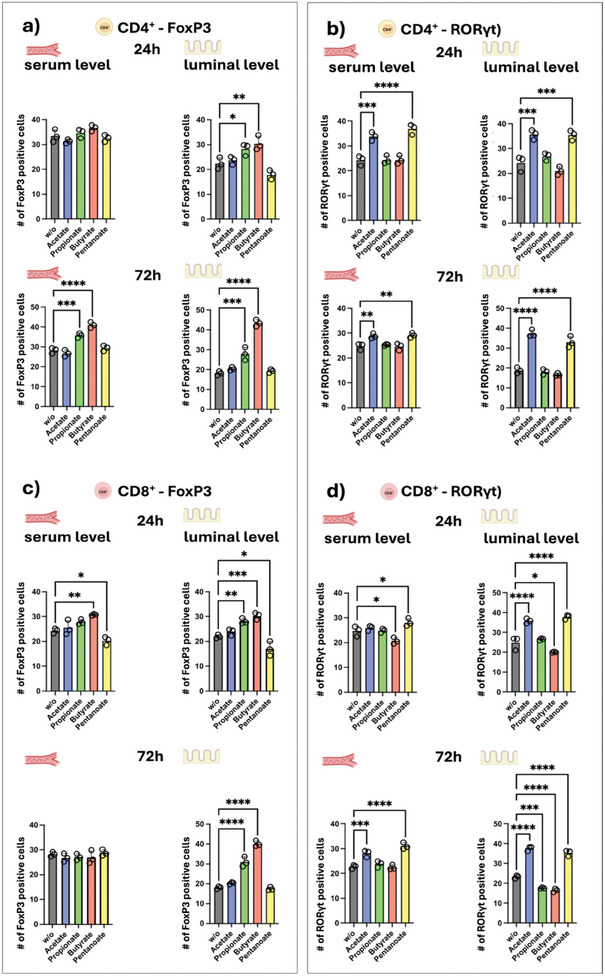
FoxP3 and RORγt expression of CD4^+^ and CD8^+^ anti‐ROR1 CAR T‐cells. CD4^+^ and CD8^+^ anti‐ROR1 CAR T‐cells were preincubated for 24 or 72 h with SCFAs at serum and luminal concentrations. Diagrams show flow cytometric analysis of the percentage of a,c) FoxP3 and b,d) RORγt positive cells. Statistical significance was determined by using a one‐way ANOVA with Tukey's multiple comparison test. Bars represent mean ± SD of 3 independent experiments (*n* = 3). *p < 0.05, **p < 0.01, ***p < 0.001, ****p < 0.0001.

However, the CAR T‐cell incubation with propionate and butyrate, induced a dose‐ and time‐dependent upregulation of FoxP3 expression in CD4^+^ and CD8^+^ CAR T‐cells, indicating a shift toward a regulatory T‐cell (T_reg_) phenotype. This anti‐inflammatory profile potentially diminishes antitumoral activity. It also resonates with previous findings, demonstrating that specific SCFAs, such as butyrate and propionate, can promote T_reg_ differentiation.^[^ ^15^ ^]^ Such effects contribute to immune homeostasis but may also impair effector functions critical for robust antitumor immunity in ACT therapies. Conversely, the observed increase of RORγt expression by the addition of acetate and pentanoate indicates the induction of a proinflammatory T‐helper 17 (Th_17_) phenotype, consistent with existing evidence that SCFAs can differentially modulate T‐cell polarization^[^ ^9^, ^33^ ^]^ (**Figures** **10**; Figure S14, Supporting Information).

**Figure 11:**
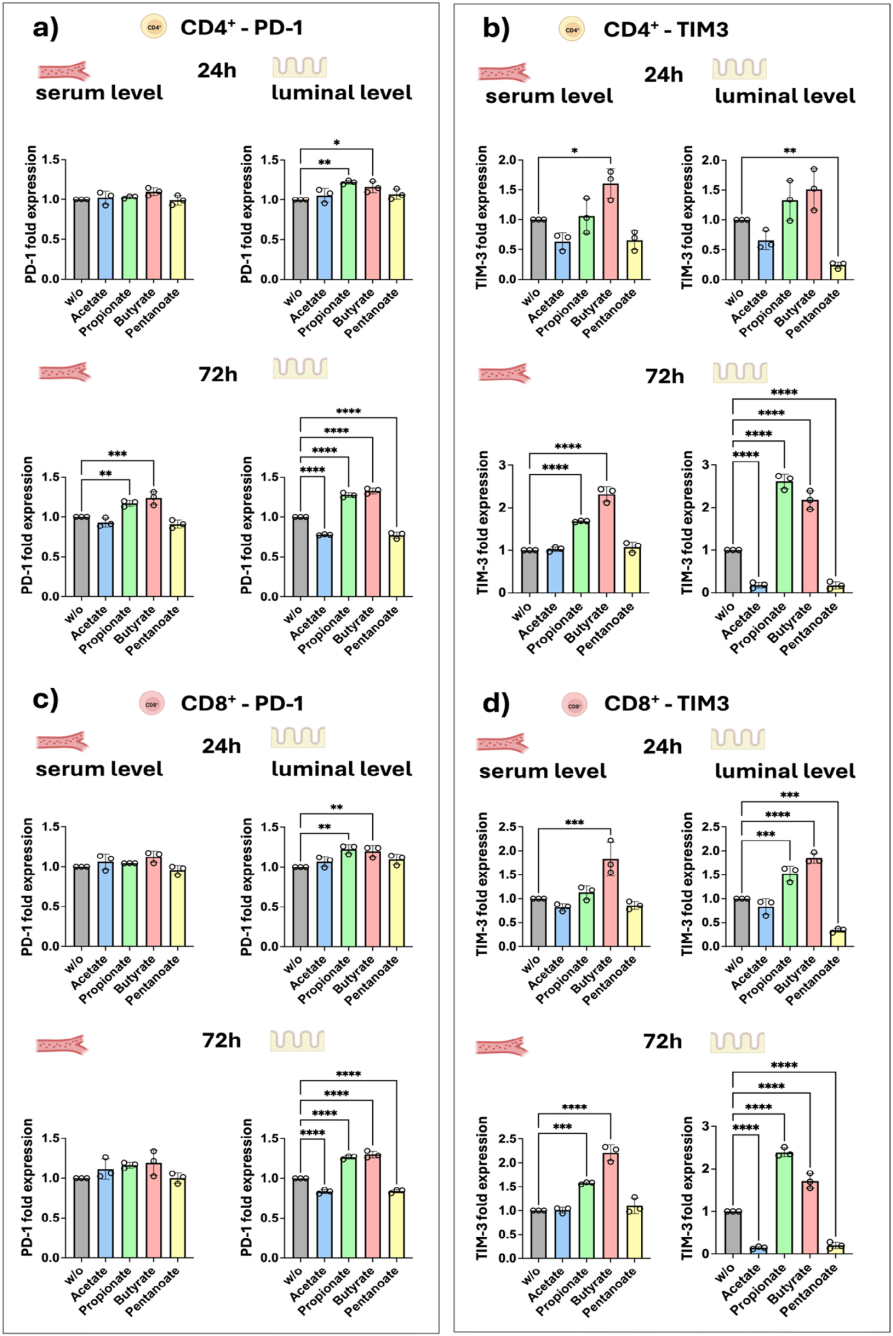
PD‐1 and TIM‐3 expression of CD4^+^ and CD8^+^ anti‐ROR1 CAR T‐cells. CD4^+^ and CD8^+^ anti‐ROR1 CAR T‐cells were preincubated for 24 or 72 h with SCFAs at serum and luminal concentrations. Diagrams show flow cytometric analysis of the fold‐change of the expression of a,c) PD‐1 and b,d) TIM‐3. Statistical significance was determined by using a one‐way ANOVA with Tukey's multiple comparison test. Bars represent mean ± SD of 3 independent experiments (*n* = 3). *p < 0.05, **p < 0.01, ***p < 0.001, ****p < 0.0001.

Our findings further reveal that stimulation with the SCFAs propionate and butyrate leads to a significant upregulation of PD‐1 and TIM‐3 in both CD4^+^ and CD8^+^ anti‐ROR1 CAR T‐cells, indicating an induction of T‐cell exhaustion^[^ ^20^ ^]^ and immune tolerance.^[^ ^21^ ^]^ This correlated with reduced CAR T‐cell cytotoxicity and a dampened proinflammatory cytokine response in our IAC model. In contrast, pentanoate treatment resulted in a downregulation of PD‐1 and TIM‐3, suggesting a potential role in maintaining CAR T‐cell activity and preventing exhaustion. Acetate did not significantly impact the expression of these markers, further underscoring the metabolite‐specific immune modulation of SCFAs (**Figure** **11**).

**Figure 12:**
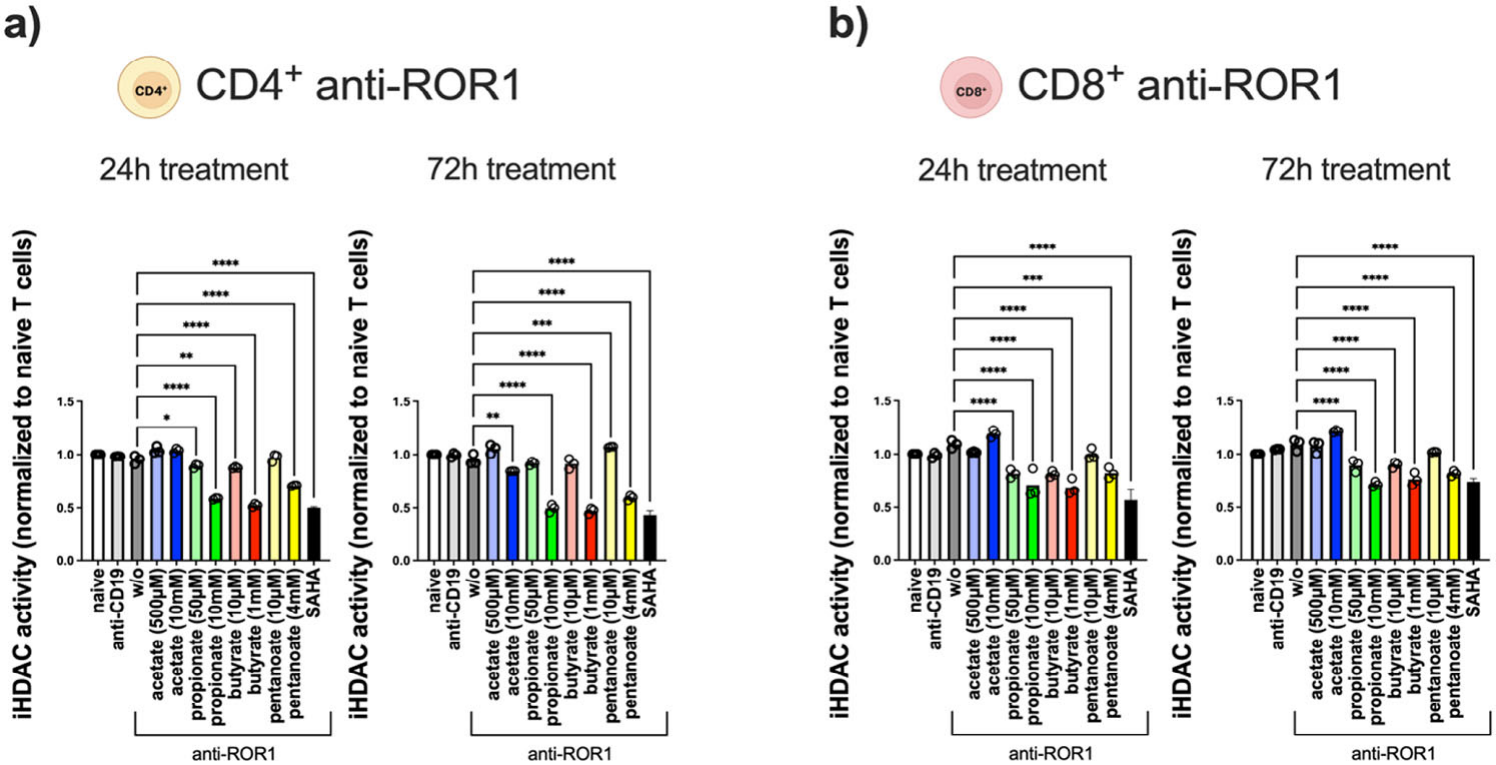
HDAC inhibition activity mediated by SCFA or SAHA in anti‐ROR1 CAR T‐cells. a) CD4^+^ and b) CD8^+^ anti‐ROR1 CAR T‐cells were pretreated with SCFA at serum and luminal level concentrations for 24 and 72 h. Statistical significance was determined using a one‐way ANOVA with Tukey's multiple comparison test. Bars represent mean ± SD of 3 independent experiments (*n* = 3) with three data points per replicate. *p < 0.05, **p < 0.01, ***p < 0.001, ****p < 0.0001.

SCFAs are also well known for modulating gene expression by inhibiting HDAC activity.^[^ ^10^, ^11^ ^]^ We, therefore, investigated whether SCFA pretreatment could impact HDAC activity in anti‐ROR1 CAR T‐cells. Our results confirmed a significant reduction in HDAC activity upon preincubation with propionate, butyrate, and pentanoate, particularly at higher luminal‐level SCFA concentrations, in both CD4^+^ and CD8^+^ CAR T‐cells after 24 and 72 h of treatment (**Figure** **12**). This finding indicates that SCFAs likely modulate epigenetic regulation in anti‐ROR1 CAR T‐cells, which may contribute to the observed reduction in their antitumor activity.

To further assess the role of HDAC activity in CAR T‐cell functionality, we used the HDAC inhibitor SAHA to determine its inhibitory effects on CAR T‐cell function. SAHA treatment did not impact cell viability (Figure S16, Supporting Information) but reduced HDAC activity similar to SCFAs (Figure 12).

**Figure 13:**
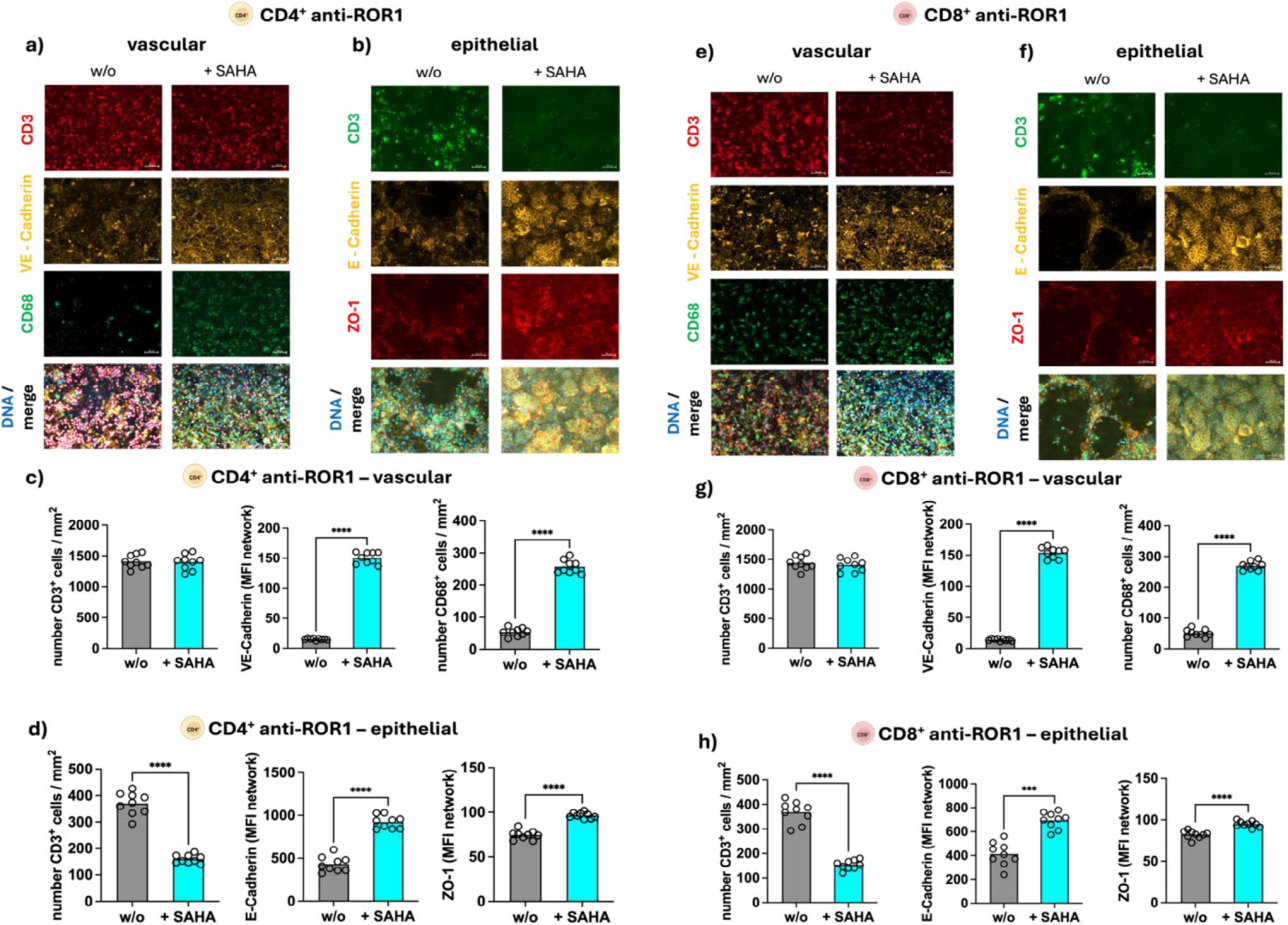
Effects of SAHA treatment (72 h) on CAR T‐cells in the IAC model. A 1:1 mix of CD4^+^ and CD8^+^ anti‐ROR1 CAR T‐cells were preincubated for 72 h with SAHA and perfused in the IAC model. a,e) Vascular side stained for CD3 (red), VE‐cadherin (yellow), CD68 (green), and merged channels including DNA (DAPI, blue). b,f) Epithelial layer stained for CD3 (green), E‐cadherin (yellow), ZO‐1 (red), and merged including DNA (DAPI, blue). c,d,g,h) The corresponding quantification. Scale bars represent 50 µm. Statistical significance was determined using a one‐way ANOVA with Tukey's multiple comparison test. Bars represent mean ± SD of 3 independent experiments (*n* = 3) with three data points per replicate. ****p < 0.0001.

Furthermore, SAHA treatment had no significant effects on the energy metabolism of CD4^+^ or CD8^+^ CAR T‐cells at the levels of acetyl‐CoA formation rate or the ADP/ATP ratio within 24 or 72 h of treatment (Figure S14, Supporting Information). However, similar to propionate and butyrate HDAC inhibition by SAHA prevented CAR T‐cell infiltration into the enterocyte layer. In addition, SAHA treatment of CAR T‐cells preserved VE‐cadherin, E‐cadherin, and ZO‐1 in ROR1‐expressing target cells, demonstrating that HDAC inhibition specifically reduces CAR T‐cell cytotoxicity. These findings were consistent across CD4^+^ and CD8^+^ CAR T‐cells and observed for 24 and 72 h preincubation before perfusion within the IAC model (Figure S17, Supporting Information; **Figure** **13**).

**Figure 14:**
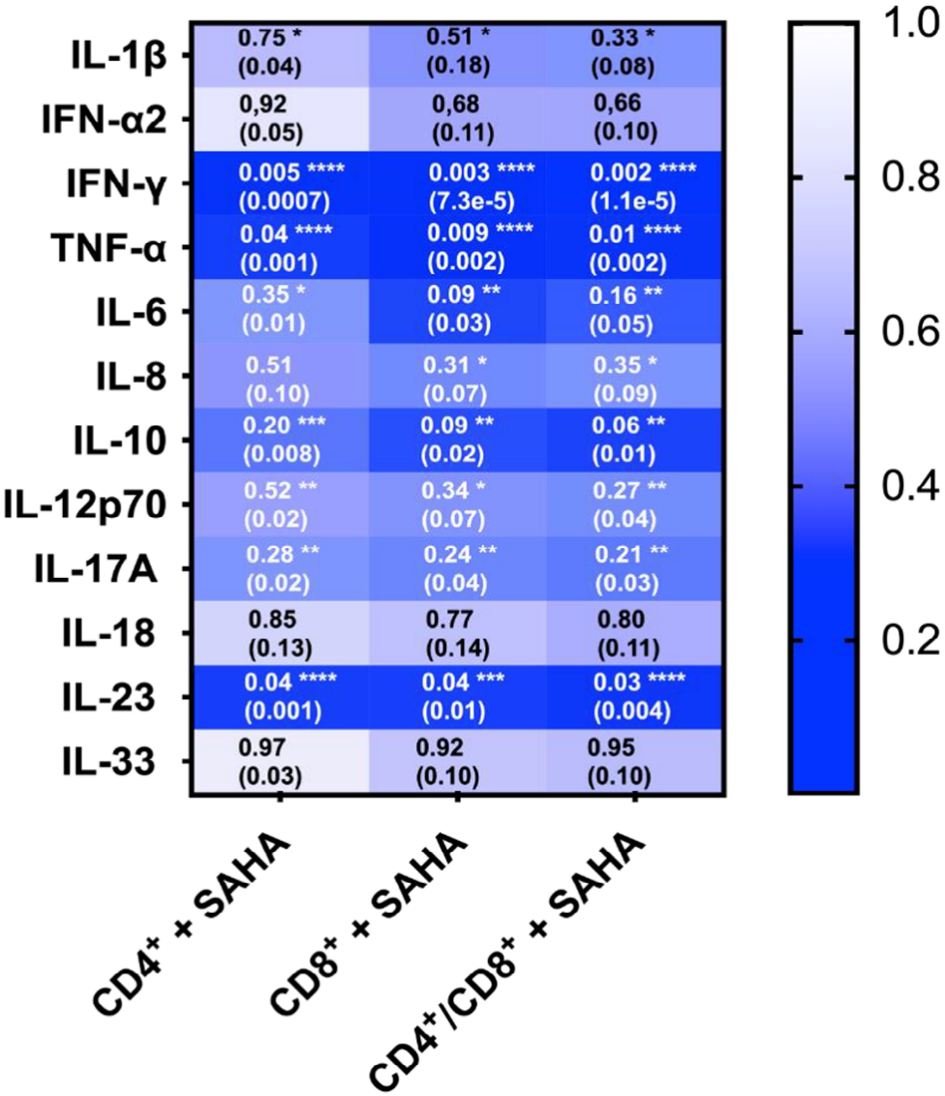
Cytokine release of the IAC model perfused with CD4^+^ and CD8^+^ anti‐ROR1 CAR T‐cells after 72 h of preincubation with SAHA. Values were normalized against an untreated control and statistical significance was determined using a one‐way ANOVA with Tukey's multiple comparison test. Values represent mean ± SD of 3 independent experiments (*n* = 3). *p < 0.05, **p < 0.01, ***p < 0.001, ****p < 0.0001.

SAHA treatment of CAR T‐cells further efficiently suppressed the release of key proinflammatory cytokines, including IL‐1β, TNF, IL‐6, IL‐8, IL‐17, and IFN‐γ, as well as other immune‐modulatory cytokines such as IL‐10, IL‐12, and IL‐23. This suppression was observed for CD4^+^, CD8^+^, and CD4^+^/CD8^+^ CAR T‐cell cocultures 72 h after SAHA pretreatment and subsequent circulation in the IAC model (**Figure** **14**).

Our findings suggest that SCFAs similar to SAHA exert their suppressive effects on CAR T‐cell activity, at least partly through epigenetic regulation via HDAC inhibition rather than through modulating cellular energy metabolism. Epigenetic changes induced by HDAC inhibition are essential for decreasing CAR T‐cell infiltration and the subsequent attack and destruction of tumor cells. Our results further underline the significant immunosuppressive effects of HDAC inhibition on cytokine production by CAR T‐cells. These findings mirror the trends observed with SCFA pretreatment and underscore the importance of epigenetic modulation in regulating CAR T‐cell effector functions within the tumor microenvironment.

## 3 Discussion

We here introduce a human microfluidic IAC‐on‐chip model for studying CAR T‐cell interaction with tumor cells, considering the effects of microbial metabolites. Unlike traditional static models, the IAC system replicates physiologically relevant shear forces, preventing artificial sedimentation of CAR T‐cells and ensuring dynamic interactions with the tumor microenvironment. This reflects in vivo conditions, where T‐cell circulation and extravasation are crucial in effective tumor infiltration. Additionally, the IAC model provides a controlled platform for high‐resolution microscopic imaging, allowing the visualization of CAR T‐cell infiltration and the assessment and quantification of their cytotoxic effects based on the alteration of tissue biomarkers of tumor cells such as apical junctional complex protein expression.

In contrast to murine models, which often exhibit interspecies variations in immune cell function and tumor microenvironment interactions, our model utilizes human‐derived immune and tumor target cells. This is particularly important for immunological studies, as species‐specific differences in cytokine signaling, antigen presentation, and T‐cell receptor interactions can significantly impact experimental outcomes.^[^ ^34^ ^]^ By incorporating human cells cultured under physiologically relevant conditions, our microfluidic IAC system provides a promising platform for evaluating CAR T‐cell interactions with the tumor microenvironment under near‐physiological conditions.

In this study, we selected ROR1 as the target antigen primarily due to its established expression in colorectal adenocarcinomas and its emerging relevance in solid‐tumor‐targeted CAR T‐cell therapies.^[^ ^35^ ^]^ We acknowledge, however, that ROR1 expression is not entirely restricted to tumor cells in our model, as evidenced by our observation of comparable ROR1 expression levels in HUVECs used within the IAC model. Consequently, we detected specific CAR T‐cell‐mediated cytotoxicity toward endothelial cells, as shown by reduced VE‐cadherin expression.

Leveraging this model, we highlight the nuanced role of SCFAs in shaping CAR T‐cell functionality within the tumor microenvironment, reflecting their dual roles as immunosuppressive and proinflammatory modulators. The immunosuppressive effects observed in our study highlight the complex balance between CAR T‐cell efficacy and the tumor microenvironment, particularly in the context of ROR1‐expressing tumors. While ROR1 serves as a promising target due to its selective expression in malignancies, our findings indicate that metabolic influences from the gut microbiota, specifically SCFAs, can critically shape CAR T‐cell functionality.

We demonstrated that butyrate and propionate drive a T_reg_‐like phenotype, characterized by increased FoxP3 and RORγt expression, reinforcing an immunosuppressive environment that may limit CAR T‐cell efficacy in solid tumors. In our study, propionate and butyrate induced a dose‐ and time‐dependent upregulation of FoxP3 in CD4^+^ and CD8^+^ CAR T‐cells, promoting a T_reg_ phenotype. This anti‐inflammatory shift, characterized by reduced antitumoral activity, is consistent with prior studies in mice that demonstrated how SCFAs like butyrate and propionate could foster T_reg_ differentiation via HDAC inhibition.^[^ ^36^ ^]^ Such effects are critical for maintaining immune homeostasis but may attenuate the effector functions necessary for effective antitumor immunity in ACT therapies. Conversely, acetate and pentanoate increased RORγt expression, which is indicative of a proinflammatory Th_17_ phenotype. This underscores the differential impact of SCFAs on T‐cell polarization and function^[^ ^15^ ^]^ as they can promote effector T‐cell phenotypes through epigenetic modulation. For instance, acetate has been shown to enhance Th17 differentiation and increase RORγt expression via HDAC inhibition and activation of the mTOR‐S6K pathway.^[^ ^15^ ^]^ Similarly, pentanoate has been highlighted as a potent inducer of Th1/Th17‐associated transcription factors such as RORγt and T‐bet. While on the other hand, it suppresses FoxP3 expression, which is critical for T_reg_ development.^[^ ^33^ ^]^ This phenotype is associated with reduced antitumoral activity, aligning with previous findings on SCFA‐induced T_reg_ differentiation.^[^ ^15^, ^37^ ^]^

This metabolic reprogramming was accompanied by a striking upregulation of PD‐1 and TIM‐3 in CD4⁺ and CD8⁺ CAR T‐cells, suggesting an SCFA‐driven induction of T‐cell exhaustion. In contrast, pentanoate was found to reduce the expression of these exhaustion markers. T‐cell exhaustion, marked by sustained PD‐1 and TIM‐3 expression, is a well‐recognized barrier to effective anticancer immunity, particularly in solid tumors where chronic antigen exposure and immunosuppressive signals contribute to a functional decline of antitumoral immune responses. PD‐1 and TIM‐3 not only inhibit CAR T‐cell activation and proliferation but also impair their cytokine production and cytotoxic responses, ultimately facilitating tumor cell immune evasion.^[^ ^38^ ^]^ The induction of these exhaustion markers by butyrate and propionate suggests a mechanism by which microbial metabolites can actively suppress CAR T‐cell function, a phenomenon that has also been observed in other immunotherapy contexts.^[^ ^20^ ^]^ Notably, PD‐1 and TIM‐3 expression correlate with poor prognosis and the formation of an immune‐tolerant tumor microenvironment, which can significantly diminish the durability and effectiveness of CAR T‐cell therapies. The differential effect of pentanoate, which downregulated PD‐1 and TIM‐3 on CAR T‐cells, indicates that SCFAs do not act uniformly but elicit opposing or distinct immune‐modulatory effects depending on the specific SCFA involved.

This is underlined by our finding that butyrate and propionate in addition exhibited a suppressive effect on CAR T‐cell infiltration into the tumor model, which was associated with reduced antitumor efficacy of CAR T‐cells. This effect is consistent with studies showing that butyrate can also inhibit cytokine release and decrease the expression of proinflammatory cytokines like TNF, IL‐6, and IFN‐γ. This decreases, in turn, further limits the antitumor efficacy of the CAR T‐cells and their ability to infiltrate tumor sites.^[^ ^21^, ^39^, ^40^, ^41^ ^]^ In a recent study, butyrate and propionate were shown to restrict the antitumor effects of T‐cells by affecting several key mechanisms related to immune activation and T‐cell functionality.^[^ ^20^, ^29^ ^]^ Butyrate restrained the induction of tumor‐specific CD8^+^ T‐cells. In mice treated with anti‐CTLA‐4, butyrate supplementation significantly reduced IFN‐γ production—a key marker of T‐cell activation and antitumor function. This indicates that butyrate can reduce the efficacy of T‐cell priming and function.^[^ ^42^ ^]^ In patients, high butyrate levels were associated with an increased frequency of T_regs_, which could dampen the immune response against tumors.^[^ ^42^ ^]^ Butyrate and propionate can also impact cellular metabolism by providing acetyl‐CoA, fueling the TCA cycle, and altering ATP production.^[^ ^43^, ^44^ ^]^ However, we have found no changes in acetyl‐CoA formation and ADP/ATP ratio related to SCFA stimulation and CAR T‐cell function alterations, indicating other CAR T‐cell inhibition mechanisms in the IAC model.^[^ ^45^ ^]^

The differential modulation of T cell differentiation by SCFAs likely reflects their ability to inhibit HDAC activity, which plays a pivotal role in epigenetic regulation of lymphocyte differentiation.

HDAC modulation can, depending on the context, lead to different outcomes, such as promoting regulatory phenotypes or inhibiting effector functions of immune cells. Propionate and butyrate can increase the acetylation of histone H3 in T‐cells, resulting in diminished activation in a concentration‐dependent manner. This includes an immunosuppressive phenotype and constraining antitumor immune response.^[^ ^36^, ^42^ ^]^ Notably, for butyrate, the timing of exposure seems critical for its inhibitory effects. Butyrate needed to be present before or early after TCR stimulation to effectively dampen T‐cell activity. This suggests that butyrate needs to act early in the activation process to modify gene expression through mechanisms, such as HDAC inhibition.^[^ ^40^ ^]^ The differential HDAC inhibitory capacity of SCFAs may explain their distinct impacts on T‐cell phenotypes.

Previous studies have reported that pentanoate can significantly increase the production of effector molecules, such as IFN‐γ and TNF, thereby boosting the antitumor activity of both CD8^+^ T‐cells and CAR T‐cells. Hereby the antitumor efficacy is improved through metabolic and epigenetic reprogramming.^[^ ^13^, ^46^, ^47^ ^]^ In our study, pentanoate had a minimal impact on CAR T‐cell activity in the IAC model. Specific cultural conditions can explain these differences. We cultured the CAR T‐cells with SCFAs for 24 or 72 h without additional CD3 and CD28 costimulation. However, in other studies, the TCR was costimulated during SCFA exposure.^[^ ^13^ ^]^ TCR costimulation is known to alter the metabolic and functional state of T‐cells significantly.^[^ ^48^ ^]^ Differentiation of T‐cells can be substantially influenced by the strength of TCR signaling, as well as the level of costimulation and antigen potency. When TCR signals are weakened, T‐cells are more likely to differentiate into FoxP3‐expressing T_regs_ instead of proinflammatory T‐cells.^[^ ^49^, ^50^, ^51^ ^]^ Thus, the strength of TCR signaling and costimulation is crucial in balancing regulatory and effector T‐cell differentiation. This also influences the shaping of the immune responses in the presence of varying SCFA types and concentrations.

This dynamic interplay between TCR signaling, costimulation, and SCFA‐mediated modulation not only governs T‐cell differentiation but also influences the broader immune response, including the extent of CAR T‐cell activation and cytokine secretion. In this context, the impact of SCFAs extends beyond immune cell programming to affect the integrity of epithelial and endothelial barriers, shaping the tumor microenvironment and the outcome of CAR T‐cell‐mediated cytotoxicity. VE‐cadherin, E‐cadherin, and ZO‐1 are critical components of endothelial and epithelial cell junctions. In our experiments, the interaction of anti‐ROR1 CAR T‐cells with ROR1‐expressing tumor cells in the IAC model was associated with the release of proinflammatory cytokines, including TNF, IL‐6, and IFN‐γ. This release correlated with the loss of these junctional proteins, confirming their validity as biomarkers for CAR T‐cell‐mediated cytotoxicity. These findings align with existing studies that have demonstrated the critical roles of cytokines such as IFN‐γ and TNF in disrupting junctional integrity and contributing to the cytotoxic effects of T‐cell‐mediated responses.^[^ ^52^ ^]^ The release of these cytokines contributes to vascular leakage, which may facilitate immune cell infiltration but also poses risks of off‐target effects such as cytokine release syndrome and local inflammation‐induced tissue damage.^[^ ^53^ ^]^ The treatment with propionate and butyrate led to a significant reduction in cytokine release by CAR T‐cells, which corresponded to the preservation of junctional protein expression in the IAC model.

Importantly, we also measured IL‐6 and IL‐8, key inflammatory mediators in the tumor microenvironment. IL‐6, known for its dual role in promoting inflammation and immune regulation, can support tumor progression by driving STAT3 activation and immune evasion mechanisms while contributing to CAR T‐cell exhaustion.^[^ ^54^ ^]^ IL‐8, a potent chemokine, is associated with the recruitment of immunosuppressive myeloid cells, particularly tumor‐associated macrophages and neutrophils, which can counteract CAR T‐cell efficacy by creating an immunosuppressive microenvironment.^[^ ^55^, ^56^ ^]^ Our study further revealed the depletion of CD68‐positive macrophages in the presence of anti‐ROR1 CAR T‐cells. While macrophages are not direct targets of CAR T‐cells, their depletion likely results from releasing cytotoxic cytokines, such as TNF, from CAR T‐cells within the tumor microenvironment.^[^ ^57^ ^]^ This finding underscores the complex interplay between immune and nonimmune cells and highlights the broader effects of CAR T‐cell activity, which is partially reflected in the IAC model. Future studies should investigate whether the observed endothelial cytotoxicity occurs in vivo and clarify its clinical significance.

Our findings emphasize the duality of SCFAs, which can act as immunosuppressive or immunostimulatory agents depending on their specific chemical properties, the immune cell subset, and the broader microenvironmental context. The contrasting effects of SCFAs on CAR T‐cells highlight the critical need for personalized approaches in ACT therapy. In contrast to propionate and butyrate, pentanoate could serve as a promising immunomodulatory agent to counteract CAR T‐cell exhaustion, providing a possible strategy to enhance CAR T‐cell persistence and functionality.^[^ ^33^ ^]^ Understanding and leveraging individual microbiota profiles could lead to more effective treatments tailored to the specific metabolic environment of each patient. This is especially relevant as different SCFAs may either bolster immune responses or attenuate them, depending on the clinical objectives. Further research is needed to clarify how microbiota‐derived metabolites can be harnessed to suppress unwanted immune activation, such as in autoimmune conditions, or enhance CAR T‐cell efficacy in cancer treatment. Our findings are thus highly relevant for cancer immunotherapy, particularly in the context of ACT approaches, as they point out the potential for specific SCFAs to either bolster or impair therapeutic efficacy. Understanding the nuanced roles of individual SCFAs and their temporal effects on CAR T‐cell phenotypes is critical for developing strategies to optimize immunometabolic conditions for ACT.

Given the growing recognition of microbiota–host interactions in shaping immune responses, these findings emphasize the need to consider metabolic and microbial influences when designing CAR T‐cell therapies for solid tumors. Targeting exhaustion pathways via immune checkpoint inhibitors may offer one strategy to restore CAR T‐cell function in the presence of SCFA‐induced immunosuppression. Alternatively, modulation of gut microbiota composition, dietary interventions, or selective metabolic inhibitors could be explored to mitigate SCFA‐driven immune exhaustion while preserving the beneficial aspects of microbiota‐derived metabolites.

Our study aligns with growing evidence from recent literature that SCFAs can significantly shape the immune response by influencing the local gut environment and systemic immune functions, particularly in the context of cancer immunotherapy.^[^ ^5^, ^9^, ^21^, ^58^ ^]^ Understanding these interactions could be critical for refining CAR T‐cell therapy approaches, particularly for emerging targets like ROR1, where metabolic influences may dictate therapeutic success. Future studies should explore how SCFAs can be synergistically combined with other metabolic modulators to enhance CAR T‐cell performance. Additionally, modifying the gut microbiota to increase the production of beneficial metabolites may represent a promising avenue for improving treatment efficacy in both hematological malignancies and solid tumors. Expanding the IAC model to incorporate patient‐specific cells, such as stem cell‐derived intestinal cells and matched stool filtrates from patients, could provide a more personalized approach for assessing CAR T‐cell efficacy in relation to microbiota‐derived metabolites in the future. Advanced organ‐on‐chip systems can help identify microbiome compositions that enhance or hinder CAR T‐cell function, thus informing strategies to modify the gut microbiota for improved therapeutic outcomes. This approach allows for tailored interventions, such as microbiota‐targeted dietary changes or probiotic supplementation, to optimize CAR T‐cell activity in individual patients and will be essential for advancing the precision and personalization of CAR T‐cell therapies.

## 4 Experimental Section

### Ethics Statement

Human peripheral blood was collected from healthy volunteers after receiving written, informed consent. This study was performed following the principles outlined in the Declaration of Helsinki. The blood donation protocol and use of blood for this study were approved by the Institutional Ethics Committee of the Jena University Hospital (permission number: 2207‐01/08). HUVECs were collected under ethical approval 2020‐1684, 3939‐12/13 after donors provided written, informed consent.^[^ ^22^ ^]^

### Cell Culture

HUVECs were isolated, expanded, and cryopreserved as previously described.^[^ ^59^ ^]^ Thawed HUVECs were seeded at 2 × 10^4^ cells cm^−2^ in Endothelial Cell Growth Medium (EC medium) MV (Promocell, Heidelberg, Germany) with a supplement mix provided by the manufacturer and 1% v/v of penicillin/streptomycin (GIBCO, Thermo Fisher Scientific, Darmstadt, Germany). The medium was exchanged twice weekly, and cells were cultured up to passage 3. Once the cells reached 80–90% confluency, they were harvested and used for subsequent experiments.

Caco‐2 cells were cultivated in C2 medium, DMEM high glucose (4.5 g L^−1^) containing 10% v/v FBS, 1% v/v Glutamax, 1% v/v sodium pyruvate, 1% v/v MEM Non‐Essential Amino Acids Solution, 0.2% v/v Holotransferin, and 0.2% v/v Gentamycin (all GIBCO, Thermo Fisher Scientific, Darmstadt, Germany). The cells were cultivated up to passage 35, and once they reached 80–90% confluency were harvested and used for experiments.

Human peripheral blood mononuclear cells (PBMCs) were isolated from healthy donors using a density gradient centrifugation.^[^ ^60^ ^]^ Whole blood collected in EDTA tubes was diluted in a 1:1 ratio with Iso‐buffer (PBS without Ca^2+^/Mg^2+^, (Capricorn Scientific, Ebsdorfergrund, Germany) 0.1% v/v BSA, 2 mm EDTA (both GIBCO, Thermo Fisher Scientific, Darmstadt, Germany)) to a final volume of 35 mL and layered on 15 mL of Histopaque density gradient medium (Sigma, Merck, Darmstadt, Germany). A density gradient centrifugation was performed at 800 g for 20 min at room temperature without a break. The separated layer of immune cells was collected, washed with Iso‐buffer, and centrifugated three times. The first centrifugation was performed at 200 g for 8 min at room temperature without break, and twice at 150 g for 8 min at room temperature with break to remove residual cells and blood components. After isolation, the PBMCs were counted and seeded at a cell density of 1.25–1.5 × 10^6^ cells cm^−2^ on a 6‐well plate in 2 mL of X‐VIVO 15 medium (Lonza, Cologne, Germany) containing 10% v/v autologous serum and 0.01% v/v of both M‐CSF and GM‐CSF (Peprotech, Thermo Fisher Scientific, Darmstadt, Germany). The cells were incubated for 1 h at 5% CO_2_ and 37 °C in a humidified incubator, washed twice with X‐VIVO medium, and cultivated for an additional 24 h before being harvested with a Lidocaine/EDTA solution (4 mg mL^−1^ in PBS without Ca^2+^/Mg^2+^, 5 mm EDTA) (both GIBCO, Thermo Fisher Scientific, Darmstadt, Germany).

### Generation of ROR1 and CD19‐Specific CAR T‐Cells

PBMCs were isolated by density gradient centrifugation from leukocyte apheresis of two different healthy donors using a separating solution with a density of 1.077 g mL^−1^ (Pancoll human (PAN Biotech)). All donors provided their written informed consent.

CD4^+^ and CD8^+^ T‐cells were then isolated from PBMCs by magnetic associated cell sorting (MACS) using the CD4^+^ or CD8^+^ human T‐cell isolation kit (Miltenyi Biotec, Bergisch Gladbach, Germany), respectively. The purity of isolated T‐cell fractions was verified by staining with fluorophore‐conjugated antibodies all from Biolegend, (San Diego, CA, USA) and 7‐AAD staining solution (Miltenyi Biotec, Bergisch Gladbach, Germany) for dead cell exclusion on an MACS Quant 10 analyzer (Miltenyi Biotec, Bergisch Gladbach, Germany). The following antibodies were used: anti‐human CD3 PE (clone UCHT1), anti‐human CD4 APC (clone RPA‐T4), and anti‐human CD8 FITC (clone SK1). T‐cells were seeded in 48‐well Costar plates (Corning, New York, USA) in CTL medium (RPMI 1640 supplemented with 1% (v/v) penicillin/streptomycin, 1× GlutaMAX‐I, 0.1% (v/v) 2‐Mercaptoethanol (all from Thermo Fisher Scientific, Darmstadt, Germany), and 10% (v/v) pooled human serum (Bavarian Red Cross Center, Wiesentheid, Germany)) and activated using Dynabeads Human T‐Activator CD3/CD28 Beads and 50 U mL^−1^ rhIL‐2 (Miltenyi Biotec, Bergisch Gladbach, Germany). The next day (day 1), two‐third of the medium was removed from all wells, and T‐cells were treated with 5 ng mL^−1^ polybrene (Merck, Darmstadt, Germany). Lentiviral particles (MOI = 3) encoding the ROR1 CAR construct (ROR1_41BB_CD3zeta_EGFRt)^[^ ^61^ ^]^ or CD19CAR construct (CD19_41BB_CD3zeta_EGFRt) were added to the cells (untransduced control: polybrene only) and centrifuged at 800 × *g* for 45 min at 32 °C. The CAR cassette comprised a single chain fragment variable (scFv) derived from the anti‐human ROR1 monoclonal antibody R12 (VH‐linker‐VL), a short IgG4 hinge spacer, 4‐1BB (CD137) and CD3ζ signaling domains in cis with a T2A element, and a truncated epidermal growth factor receptor (EGFRt) as detection and selection marker. Afterward, cells were incubated for 4 h at 5% CO_2_ and 37 °C. Then, CTL medium supplemented with 50 U mL^−1^ rhIL‐2 was added to all wells, and cells were fed every other day by removing half of the medium from each well and adding CTL supplemented with 100 U mL^−1^ rhIL‐2 (final conc. 50 U mL^−1^ rhIL‐2). On day 7, CD3/CD28 beads were magnetically removed and transduction efficacy was analyzed by staining with fluorophore‐conjugated antibodies all from Biolegend (BioLegend, San Diego, CA, USA) and 7 AAD staining solution (Miltenyi Biotec, Bergisch Gladbach, Germany) for dead cell exclusion on an MACS Quant 10 (Miltenyi Biotec, Bergisch Gladbach, Germany). The following antibodies were used: anti‐human EGFR Alexa Fluor 488 (clone AY13), anti‐human CD4 APC (clone RPA T4), and anti‐human CD8 PacificBlue (clone SK1). On day 9, CAR‐modified (that is, EGFRt‐positive) T‐cells were enriched by MACS using an in‐house biotinylated anti‐EGFR antibody (Cetuximab, Eli Lilly and Company, Indianapolis, IN, USA) and anti‐Biotin Microbeads (Miltenyi Biotec, Bergisch Gladbach, Germany). The purity of enriched CAR‐modified T‐cells was analyzed as described above. On the following day, enriched CAR‐modified as well as untransduced T‐cells were subjected either to an antigen‐independent expansion protocol using irradiated CD19^+^ feeder cells as well as irradiated third‐party donor PBMCs (ROR1‐specific CAR T‐cells) or to an antigen‐dependent expansion protocol using irradiated CD19^+^ feeder cells (CD19‐specific CAR T‐cells). 14 days later, the purity of expanded CAR‐modified T‐cells was analyzed as above. Then, cells were counted using a Countess Counting II FL Device (Thermo Fisher Scientific, Darmstadt, Germany) and cryopreserved at 10 million cells per mL in Cryo SFM freezing medium (Promocell, Heidelberg, Germany).

### CAR T‐Cell Cultivation and Stimulation

The immune cells were thawed and cultivated overnight in RPMI 1640 containing 10% v/v platelet‐poor human plasma, 1% v/v penicillin/streptomycin mix, 1% v/v HEPES, 1% v/v GlutaMax, 0.1% v/v 2‐Mercaptoethanol (all GIBCO, Thermo Fisher Scientific, Darmstadt, Germany), and subsequently treated with SCFAs (Sodium acetate, sodium propionate, sodium butyrate, sodium pentanoate), SAHA (all Sigma, Merck, Darmstadt, Germany) or left untreated for 24 or 72 h at 5% CO_2_ and 37 °C in a humidified incubator. Prior to circulation in the IAC model, the cells were collected and resuspended in fresh EC medium containing supplements, autologous serum, and 1% v/v penicillin/streptavidin mix (**Table** **1**).

**Table 1:** SCFA concentrations used for stimulation.

	Luminal levels [mm]	Serum levels [µm]
Acetate	10	500
Propionate	10	50
Butyrate	1	10
Pentanoate	4	10

### Biochip Fabrication

The biochips BC002 (Dynamic42 GmbH, Jena, Germany) consisted of a polybutylene terephthalate body, forming an upper and lower chamber, divided by a 12 µm thick polyethylene terephthalate membrane with a pore density of 1 × 10^5^ pores cm^−2^ and a pore diameter of 8 µm (TRAKETCH Sabeu, Radeberg, Germany). The membrane's cell culture area comprises 2.18 cm^2^ for the upper chamber and 1.62 cm^2^ for the lower chamber.^[^ ^62^ ^]^

### Intestinal Adenocarcinoma‐on‐Chip Assembly

Before cell seeding, biochips were sterilized with 70% ethanol (VWR International GmbH, Darmstadt, Germany) and washed twice with MilliQ H_2_O (Millipore, Merck, Darmstadt, Germany). The membrane was coated using collagen IV (Sigma, Merck, Darmstadt, Germany) at a concentration of 0.5 mg mL^−1^. HUVEC cells were seeded at a density of 0.35 × 10^5^ cm^−2^ in EC medium with supplement and 1% v/v penicillin/streptavidin mix into the upper chamber of the biochip. The cells were incubated at 5% CO_2_ and 37 °C in a humidified incubator with a daily medium exchange. After 48 h, the previously isolated PBMC‐derived monocytes were seeded on top of the HUVEC cells at a density of 0.85 × 10^5^ cm^−2^ in EC medium containing supplement, autologous serum, and 1% v/v penicillin/streptavidin mix, and then cultivated for an additional 24 h. Subsequently, Caco‐2 cells were seeded on the opposite side of the membrane in the lower chamber of the biochip at a cell density of 0.42 × 10^6^ cm^−2^ in C2 medium. The biochips were incubated upside down for 24 h to facilitate cell attachment to the membrane.

After the static build‐up of the model, the biochips were connected to a peristaltic pump (ISMATEC, Wertheim, Germany) by attaching microfluidic medium reservoirs (microfluidic ChipShop, Jena, Germany) and platinum‐cured silicon tubing (Dynamic42 GmbH, Jena, Germany) to the inlets of both chambers. The chips were perfused with circular flow in both chambers at 50 µL min^−1^, matching shear stress rates of 0.013 dyn cm^−2^ (0.0013 Pa) in the top chamber and 0.006 dyn cm^−2^ (0.0006 Pa) in the bottom chamber.^[^ ^62^ ^]^

The IAC models were perfused for four days before initiating (CAR) T‐cell perfusion. 24 h before the addition of the (CAR) T‐cells, 100 ng mL^−1^ LPS (Sigma, Merck, Darmstadt, Germany) was added to the C2 medium on the Caco‐2 side. To perfuse the immune cells, the circular perfusion was switched to linear perfusion to avoid bursting of the (CAR) T‐cells passing by the peristaltic pump. The vascular layer was perfused with 1 × 10^6^ (CAR) T‐cells, with and without pretreatment in 3 mL of EC medium containing supplement, autologous serum, and 1% v/v penicillin/streptavidin mix, over the duration of 1 h before switching back to circular perfusion.

### Immunofluorescence Staining

After 24 h of T‐cell perfusion, the biochips were disconnected from the pumps. Both chambers were washed twice with cold PBS with Ca^2+^/Mg^2+^ (Capricorn Scientific, Ebsdorfergrund, Germany), and then fixed with ice‐cold methanol (Carl Roth, Karlsruhe, Germany) for 15 min at −20 °C. After fixation, the chips were washed again with PBS, containing Ca^2+^/Mg^2+^, and stored at 4 °C until immunofluorescence staining.

Before staining the cells, the membranes were removed from the biochip using a scalpel. Membranes were incubated in permeabilization and blocking solution (0.1% v/v saponin (Sigma, Merck, Darmstadt, Germany) and 3% v/v of normal donkey serum (BIOZOL, Eching, Germany) in PBS with Ca^2+^/Mg^2+^) for 30 min at room temperature. Cellular markers were stained with primary antibodies against VE‐cadherin (R&D systems, MN, USA), E‐cadherin (Sigma, Merck, Darmstadt, Germany), ZO‐1 (Invitrogen, Thermo Fisher Scientific, Darmstadt, Germany), CD3 (Abcam, Cambridge, United Kingdom), CD68 (Invitrogen, Thermo Fisher Scientific, Darmstadt, Germany), and Ki‐67 (BD Bioscience, Heidelberg, Germany). The antibodies were diluted 1:100 in staining solution (0.1% v/v saponin and 3% v/v of normal donkey serum in PBS with Ca^2+^/Mg^2+^) and incubated overnight at 4 °C. On the next day, the membranes were washed three times in washing solution (0.1% v/v saponin in PBS with Ca^2+^/Mg^2+^) before incubating them with the corresponding secondary antibodies (all Jackson ImmunoResearch, St. Thomas' Place, United Kingdom) diluted 1:200 and DAPI (Invitrogen, Thermo Fisher Scientific, Darmstadt, Germany) diluted 1:500 in staining solution for 30 min at room temperature. After three additional washing steps in washing solution, the membranes were mounted in fluorescence mounting medium (Sigma, Merck, Darmstadt, Germany) on glass slides (VWR International GmbH, Darmstadt, Germany) and covered by a glass coverslip (VWR International GmbH, Darmstadt, Germany).

### Image Acquisition and Analysis

Immunofluorescence z‐stack images were obtained with an Axio Observer 5, an ApoTome for optical sectioning, and a 20× objective, NA 0.8 all from Carl Zeiss Microscopy GmbH (Jena, Germany). The settings for excitation and emission wavelengths, as well as the exposure times, were as follows: AF647 (ex. 653 nm, em. 668 nm, 1000 ms), Cy3 (ex. 548 nm, em. 561 nm, 500 ms), EGFP (ex. 488 nm, em. 509 nm, 190 ms), DAPI (ex. 353 nm, em. 465 nm, 30 ms), and Brightfield (20 ms). Z‐stacks were imaged from the top of the sample to the membrane, with a thickness of 1.5 µm per layer, to ensure complete imaging of the whole tissue layer. Three separate images were taken per condition and staining panel. For the image quantification predefined modules of the analysis software CellProfiler^[^ ^63^ ^]^ were used to create a specific analysis pipeline for each marker.

### Flow Cytometry

All flow cytometric measurements were performed on a flow cytometer LSRFortessa (BD Bioscience, Heidelberg, Germany). The staining of the markers, to analyze the T‐cell phenotypes and the ROR1 expression, was performed according to the manufacturer's protocols. All dyes, antibodies, and chemicals were obtained from Miltenyi Biotec (Bergisch Gladbach, Germany). To quantify the expression level of ROR1 in HUVEC and Caco‐2 cells, the cells were stained with ROR1‐APC antibody. For the phenotyping, the cells were first stained for the cell surface marker CD25‐VioBright V423. After fixation and permeabilization, cells were stained with the transcription factor staining buffer set for the intracellular transcription factors FoxP3‐PE and RORγt‐APC. As positive control differentiated T‐cells expressing FoxP3 and RORγt were used.^[^ ^64^ ^]^

To assess cell viability, cells were stained with 7AAD (Invitrogen, Thermo Fisher Scientific, Darmstadt, Germany) in a 1:250 dilution in Iso‐buffer (PBS without Ca^2+^/Mg^2+^ (Capricorn Scientific, Ebsdorfergrund, Germany) 0.1% v/v BSA, 2 mm EDTA (both GIBCO, Thermo Fisher Scientific, Darmstadt, Germany)).

### Cytokine Profiles

Cytokines from the supernatant of the IAC model were measured using the LEGENDplex Human Inflammation Panel 1 (13‐plex) assay (BioLegend, San Diego, CA, USA) according to the manufacturer's manual. Supernatants were collected 24 h post immune cell perfusion, centrifuged to remove cell debris, and stored at −80 °C until measurement. Samples were measured using a flow cytometer LSRFortessa (BD Bioscience, Thermo Fisher Scientific, Darmstadt, Germany). The resulting data were analyzed using the LEGENDplex Data Analysis Software.

### Metabolic Assays

All assays were performed according to the manufacturer's protocol with (CAR) T‐cells pretreated for 24 or 72 h with or without SCFA or SAHA. In short, for the Acetyl‐Coenzyme A Assay Kit (Sigma, Merck, Darmstadt, Germany) the cells were lysed, the supernatant was collected and diluted 1:5 in assay buffer in separate wells of a black 96‐well flat bottom plate (Greiner Bio‐One GmbH, Frickenhausen, Germany). To prepare each sample, 10 µL of acetyl‐CoA Quencher was added to each well and incubated for 5 min at room temperature, followed by the addition of 2 µL Quench Remover and another 5 min of incubation at room temperature. After sample preparation, 50 µL of reaction mix was added to the respective wells and incubated for 10 min at 37 °C before measuring the fluorescence (ex. 535 nm, em. 587 nm) in a plate reader (Tecan Trading AG, Männedorf, Switzerland).

For the ADP/ATP Ratio Assay Kit (Sigma, Merck, Darmstadt, Germany), 10 000 cells in 10 µL assay buffer were added to each well of a white 96‐well plate (Greiner Bio‐One GmbH, Frickenhausen, Germany). After adding 90 µL of ATP reagent mix to each well, the plate was incubated for 1 min at room temperature, and luminescence was measured for the first time (Value 1). The second luminescence measurement (Value 2) was performed after an additional 10 min of incubation at room temperature. For the third measurement (Value 3), 5 µL ADP reagent mix was added to each well and the plate was incubated for another minute. To calculate the ADP/ATP ratio, the following formula was applied: (Value 3 − Value 2)/Value 1.

For the HDAC‐Glo I/II Assay (Promega, Walldorf, Germany), 10 000 cells per well were used in a volume of 100 µL serum‐free medium in a white 96‐well plate (Greiner Bio‐One GmbH, Frickenhausen, Germany). 100 µL of HDAC‐Glo Reagent Mix was added to each well, and the plate was incubated for 30 min at room temperature before measuring luminescence.

### Statistical Analysis

Statistical analysis of biological readouts was performed using GraphPad Prism v10.2.2 (GraphPad Software, La Jolla, CA, USA). For the comparison of multiple conditions, a one‐way ANOVA with Tukey's multiple comparison test was used. Statistical significance was included in the figures as follows: *p < 0.05, **p < 0.01, ***p < 0.001, ****p < 0.0001. A *p*‐value < 0.05 was considered statistically significant.

## Conflict of Interest

A.S.M. holds equity in Dynamic42 GmbH. A.S.M. consults Dynamic42 GmbH. M.H. is an inventor of patent applications and has been granted patents related to CAR technology, licensed in part to industry. M.H. is a cofounder and equity owner of T‐CURX GmbH, Würzburg. M.H. receives speaker honoraria from BMS, Janssen, Kite/Gilead, and Novartis, as well as research support from BMS. The other authors declare no conflict of interest.

## Author Contributions

V.D.W. performed experiments and analyzed data. A.F. contributed to flow cytometry measurement and analysis. M.A. and M.H. provided CAR T‐cells and untransduced control T‐cells. A.S.M. and P.H. conceived the project, obtained the funding, and supervised the research. V.D.W. and A.S.M. wrote the manuscript. All authors have read and approved the manuscript in its current form and agreed with its submission.

## Supporting information



Supporting Information

## Data Availability

The data that support the findings of this study are available from the corresponding author upon reasonable request.
