# Supplementary material for: Short‐Chain Fatty Acids Modulate Anti‐ROR1 CAR T‐Cell Function and Exhaustion in an Intestinal Adenocarcinoma‐on‐Chip Model
Source: Adv Healthc Mater. 2025 Apr 18;14(13):2405003. doi: 10.1002/adhm.202405003 (PMC12083441; doi:10.1002/adhm.202405003)
Supplement: Supplementary file 1 — Supporting Information [file ADHM-14-0-s001.docx]

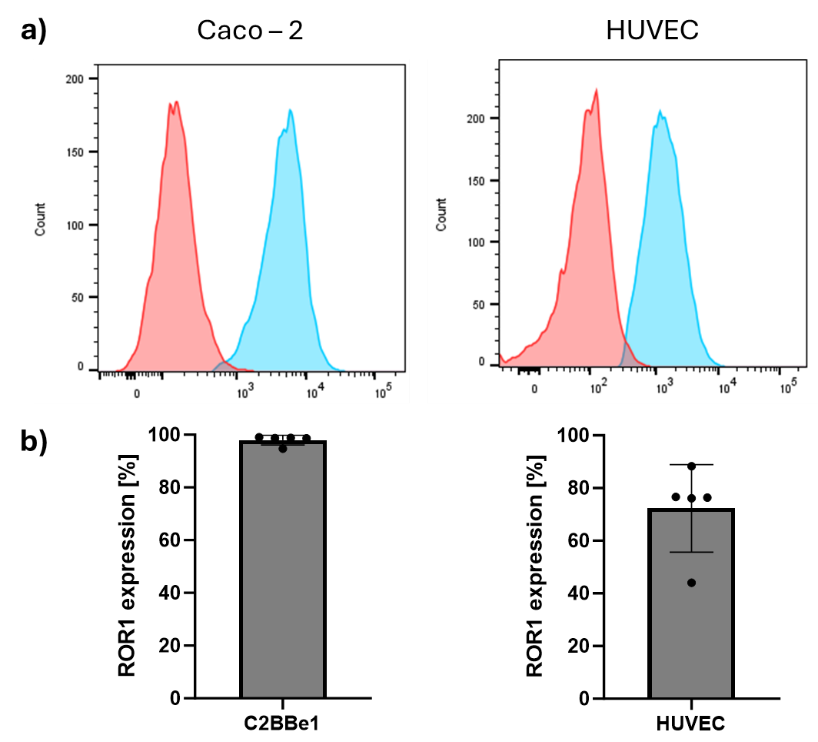


**Supplementary Figure. 1** **Expression of ROR1 on Caco-2 cells and HUVEC.** a) Representative overlay plot of ROR1 expression (blue diagram) compared to isotype control antibodies (red diagram) and b) the corresponding quantification of the percentage of ROR1 positive cells.


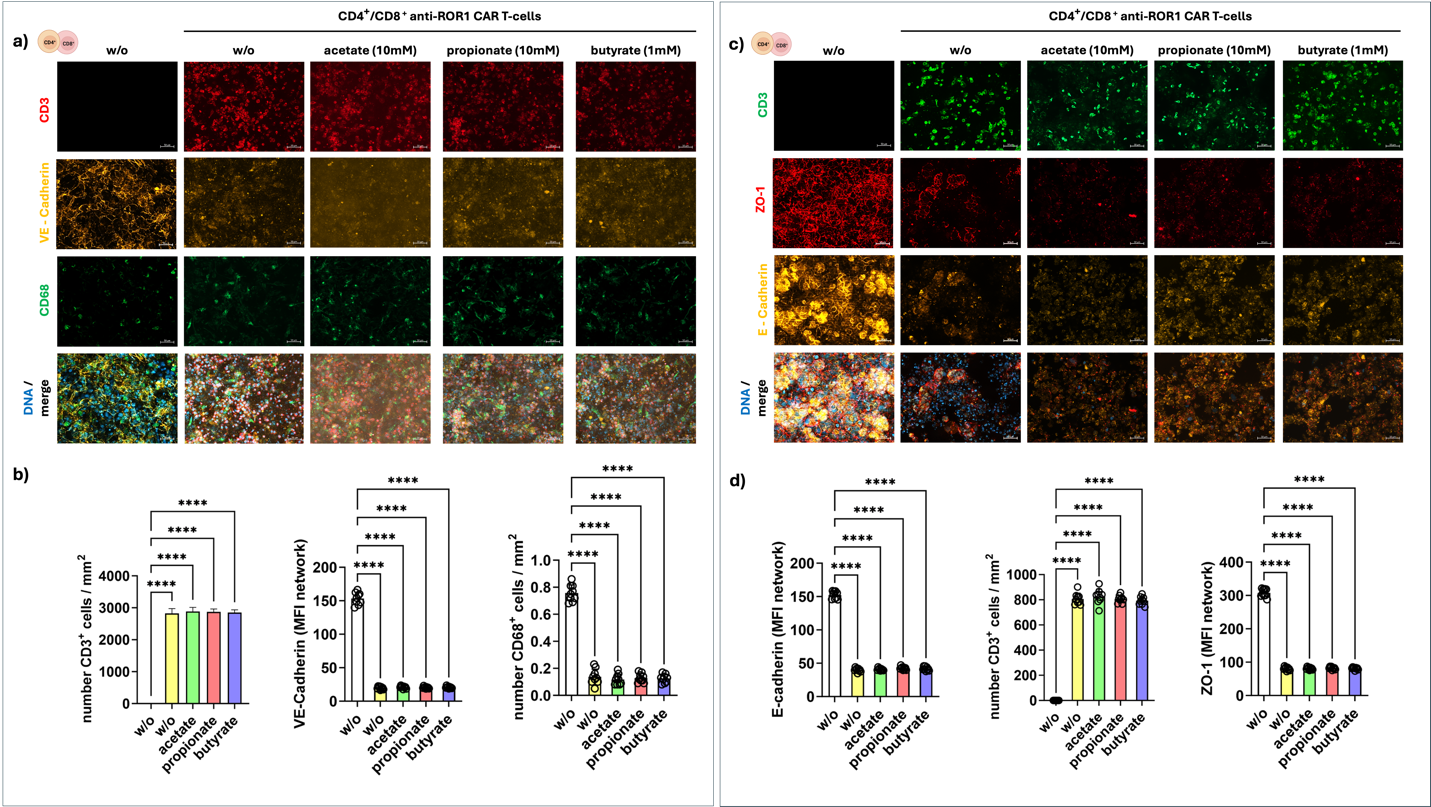


**Supplementary Figure 2.** **Perfusion of the IAC model perfused with combined CD4^+^ and CD8^+^ anti-ROR1 CAR T-cells and SCFAs.** Anti-ROR1 CAR T-cells were not pre-incubated but perfused directly in the model with SCFAs. (a) Vascular compartment: CAR T-cells stained in CD3 (red), VE-Cadherin (yellow) marks endothelial cell junctions, and CD68 (green) identifies macrophages. (c) Epithelial compartment: ZO-1 (red) highlights tight junctions, E-Cadherin (yellow) marks adherens junctions, and CAR T-cells with CD3 (green). (b-d) Quantification of biomarkers. Data are presented as mean ± SD. n = 3 independent experiments. Scale bars represent 50 µm. Statistical significance was assessed using one-way ANOVA with Tukey's multiple comparison test. **** p < 0.0001


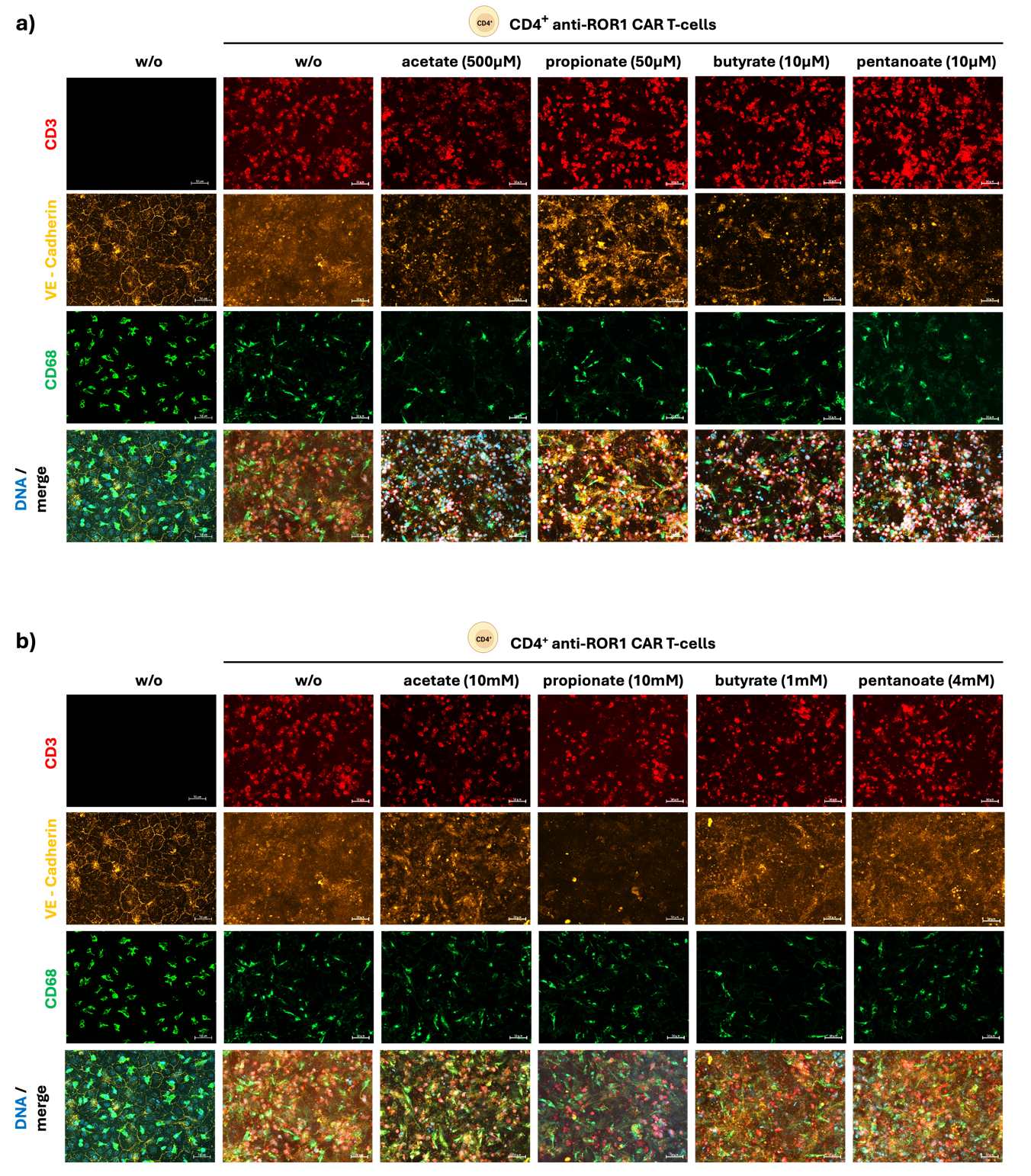


**Supplementary Figure 3.** **Vascular cell layer of the IAC model perfused with CD4^+^** **anti-ROR1 CAR T-cells.** CD4^+^ anti-ROR1 CAR T-cells were preincubated for 24 hours with a) serum-level SCFA concentrations or b) luminal-level SCFA concentrations before perfusion in the IAC model. The cells were stained for CD3 (red), VE-Cadherin (yellow), CD68 (green), and merged channels including DNA (DAPI, blue). Scale bars represent 50 µm.


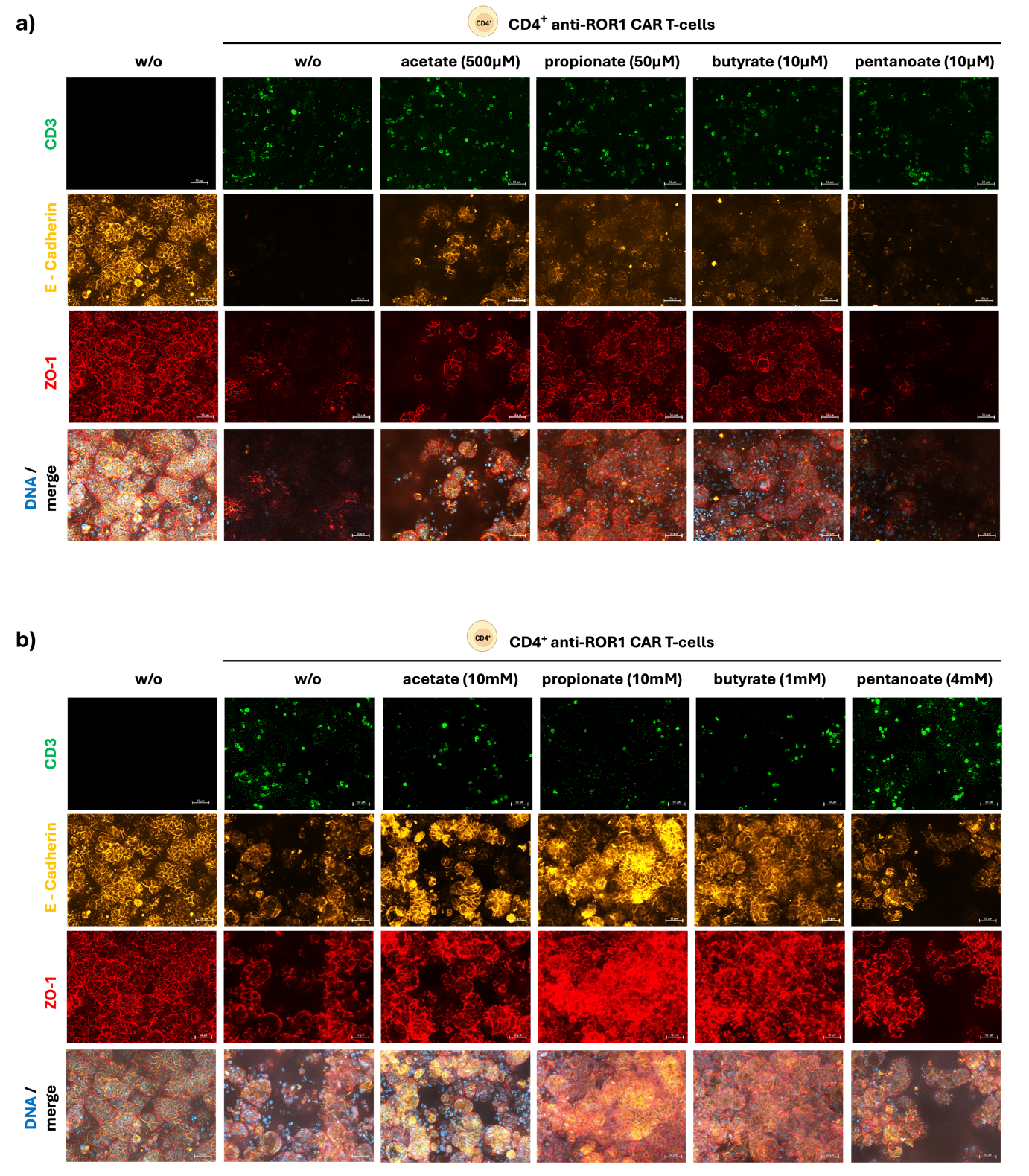


**Supplementary Figure 4.** **Epithelial cell layer of IAC model perfused with CD4^+^ anti-ROR1 CAR T-cells.** CD4^+^ anti-ROR1 CAR T-cells were preincubated for 24 hours with a) serum-level SCFA concentrations or b) luminal-level SCFA concentrations before perfusion in the IAC model. The cells were stained for CD3 (green), E-Cadherin (yellow), ZO-1 (red), and merged including DNA (DAPI, blue), and b) and d) the corresponding quantification. Scale bars represent 50 µm.


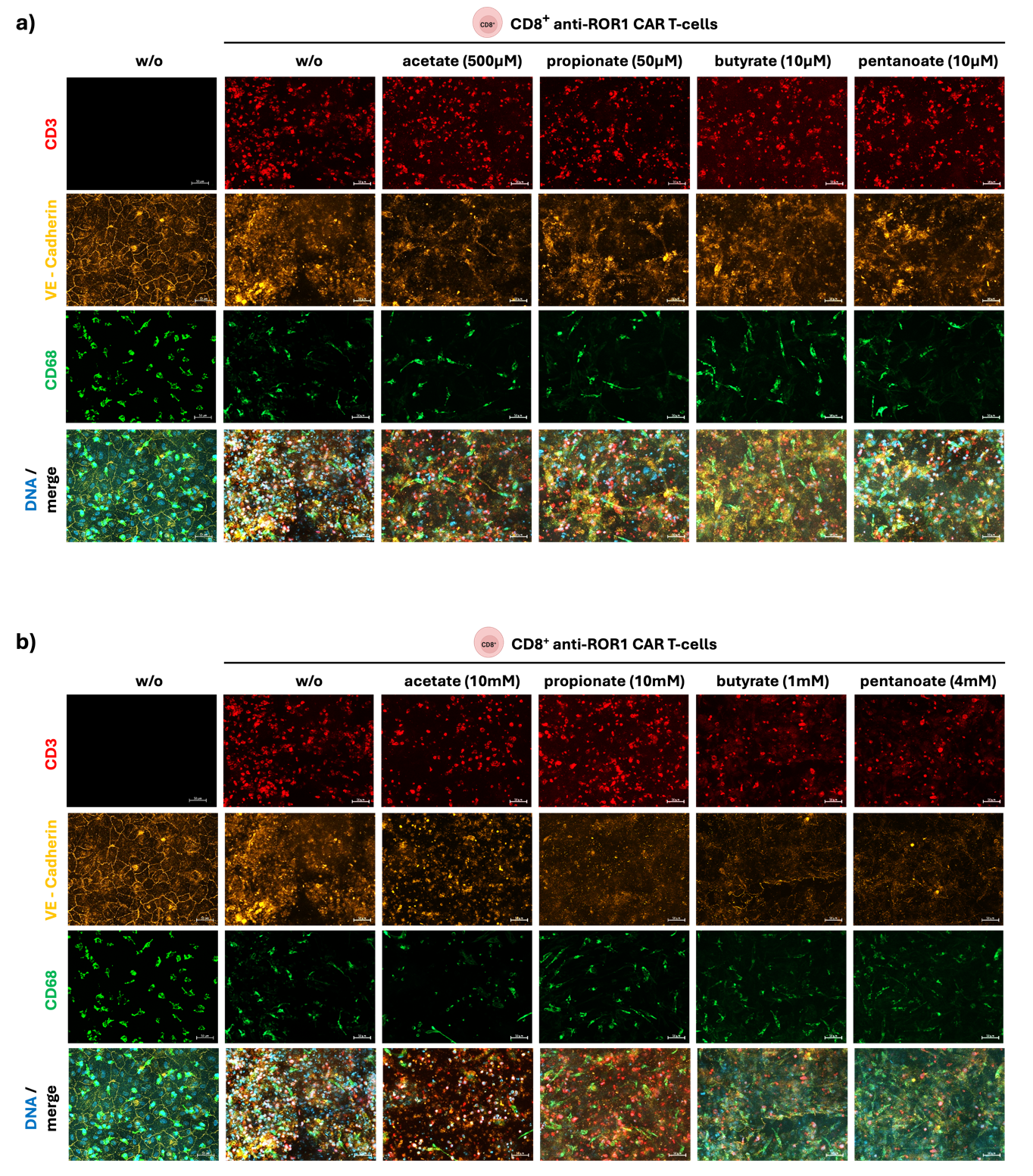


**Supplementary Figure 5.** **Vascular cell layer of the IAC model perfused with CD8^+^ anti-ROR1 CAR T-cells.** CD8^+^ anti-ROR1 CAR T-cells were preincubated for 24 hours with a) serum-level SCFA concentrations or b) luminal-level SCFA concentrations before perfusion in the IAC model. The cells were stained for CD3 (red), VE-Cadherin (yellow), CD68 (green), and merged channels including DNA (DAPI, blue). Scale bars represent 50 µm.

**
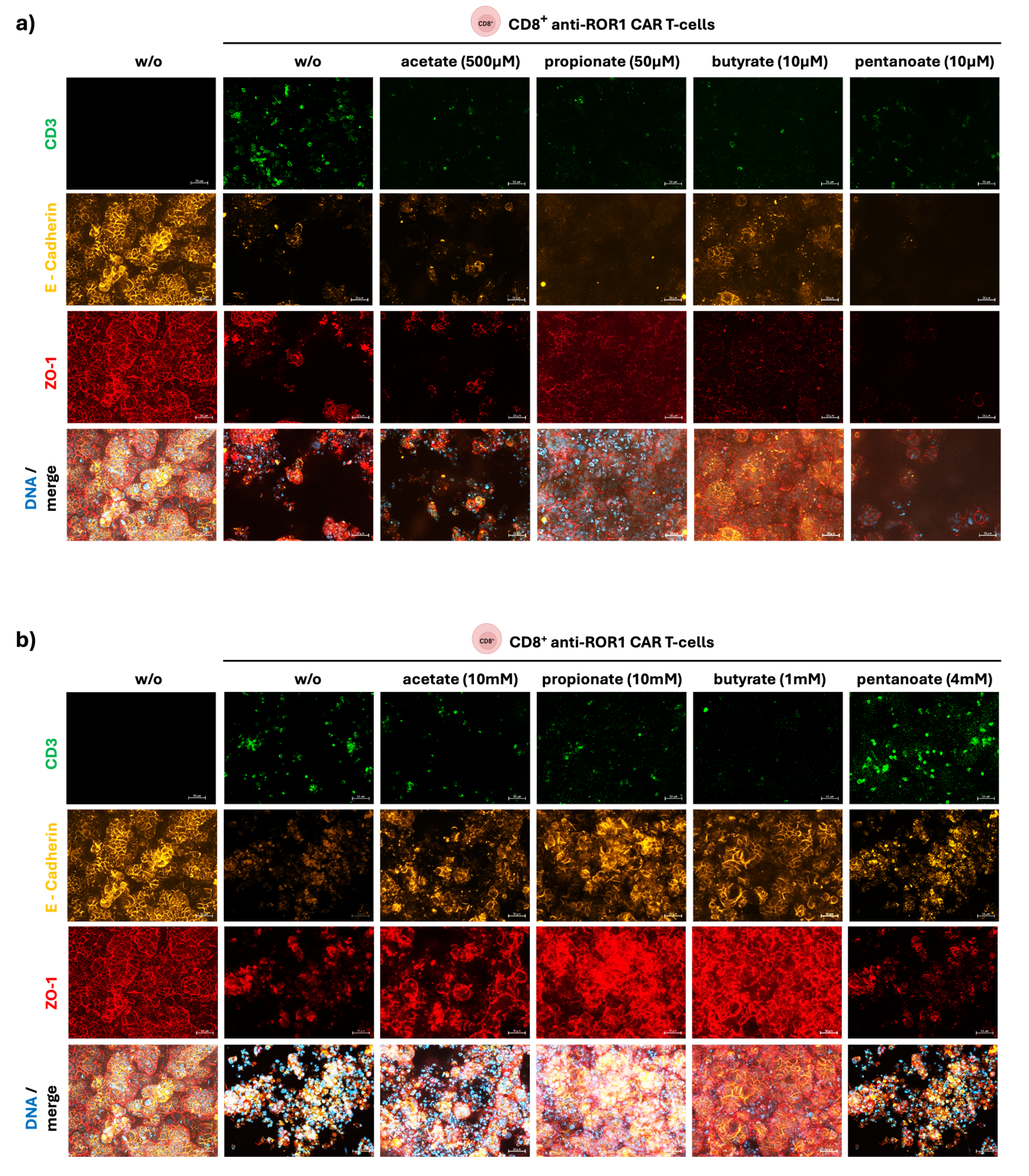
**

**Supplementary Figure 6.** **Epithelial cell layer of IAC model perfused with CD8^+^ CAR T-cells against ROR1.** CD8^+^ anti-ROR1 CAR T-cells were preincubated for 24 hours with a) serum-level SCFA concentrations or b) luminal-level SCFA concentrations before perfusion in the IAC model. The cells were stained for CD3 (green), E-Cadherin (yellow), ZO-1 (red), and merged including DNA (DAPI, blue), and b) and d) the corresponding quantification. Scale bars represent 50 µm.


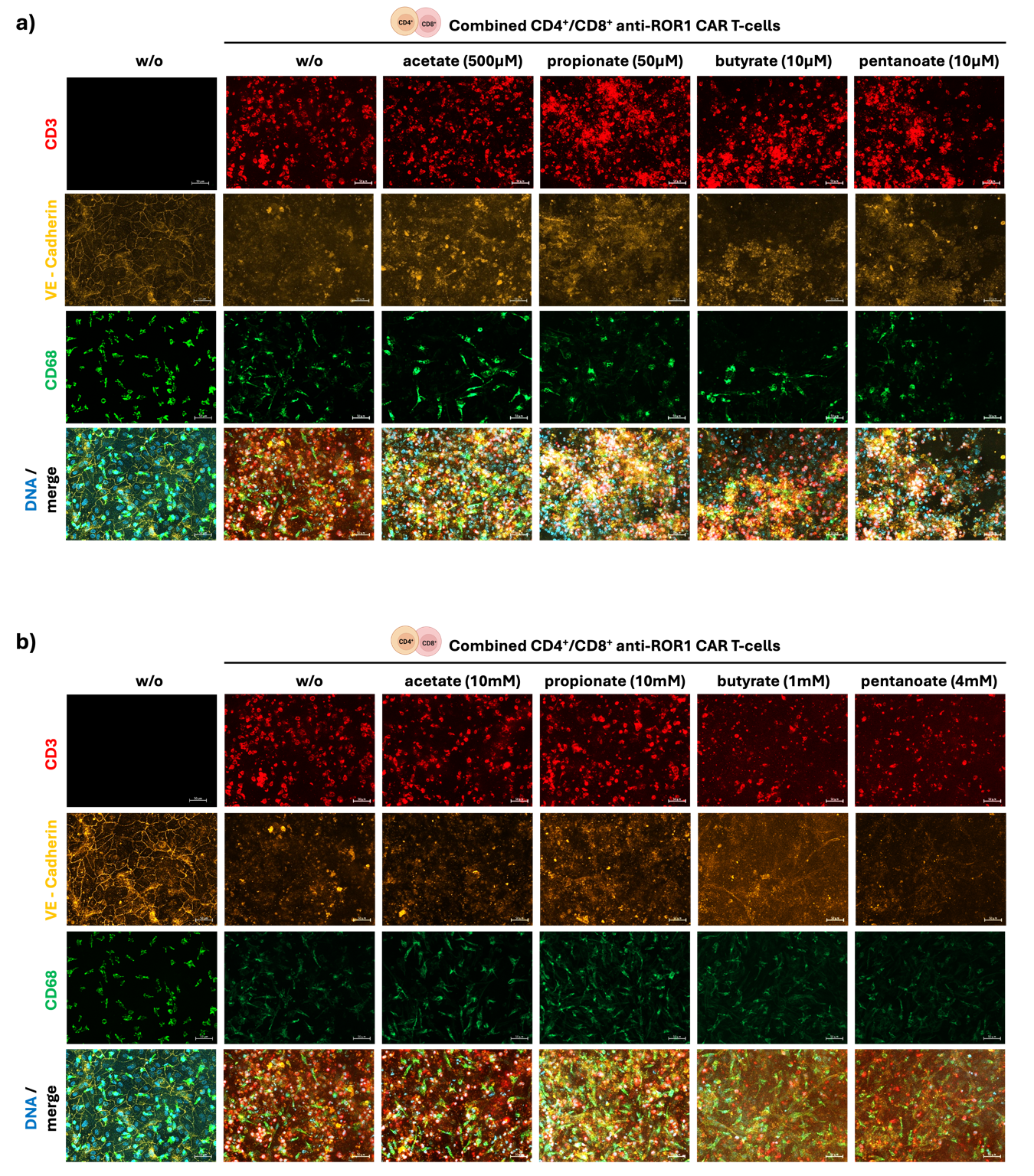


**Supplementary Figure 7.** **Vascular cell layer of the IAC model perfused with combined CD4^+^ and CD8^+^ anti-ROR1 CAR T-cells.** A mix of CD4^+^ and CD8^+^ anti-ROR1 CAR T-cells (1:1) was preincubated for 24 hours with a) serum-level SCFA concentrations or b) luminal-level SCFA concentrations before perfusion in the IAC model. The cells were stained for CD3 (red), VE-Cadherin (yellow), CD68 (green), and merged channels including DNA (DAPI, blue). Scale bars represent 50 µm.


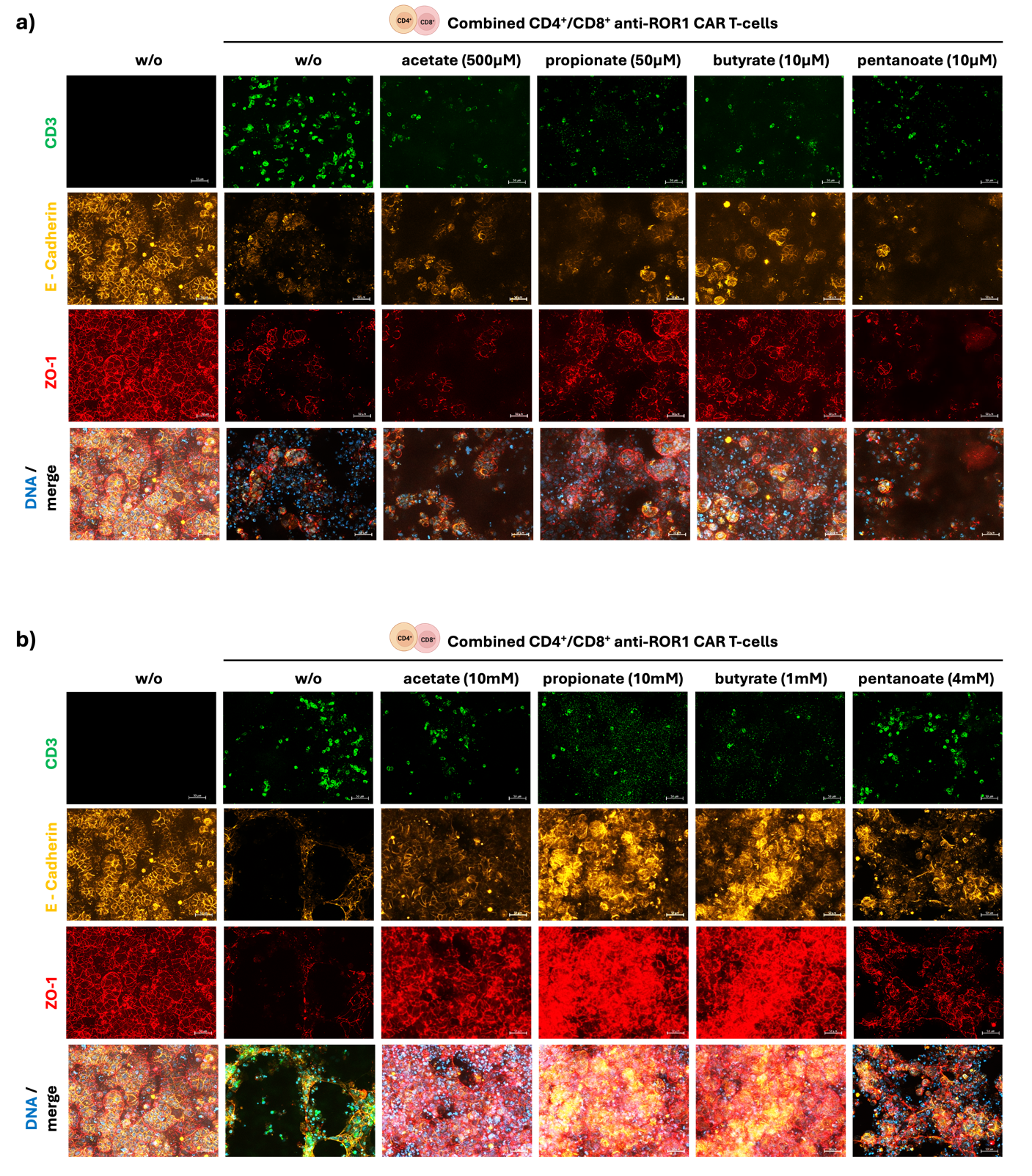


**Supplementary Figure 8.** **Epithelial cell layer of IAC model perfused with combined CD4^+^ and CD8^+^ anti-ROR1 CAR T-cells****.** A mix of CD4^+^ and CD8^+^ anti-ROR1 CAR T-cells (1:1) was preincubated for 24 hours with a) serum-level SCFA concentrations or b) luminal-level SCFA concentrations before perfusion in the IAC model. The cells were stained epithelial layer stained for CD3 (green), E-Cadherin (yellow), ZO-1 (red), and merged including DNA (DAPI, blue) and b) and d) the corresponding quantification. Scale bars represent 50 µm.

Further, preincubation of acetate, propionate, or butyrate for 24 hours, but not preincubation with pentanoate suppressed T-cell infiltration of untransduced control T-cells at serum-level concentrations (Supplementary Information Figure 9).


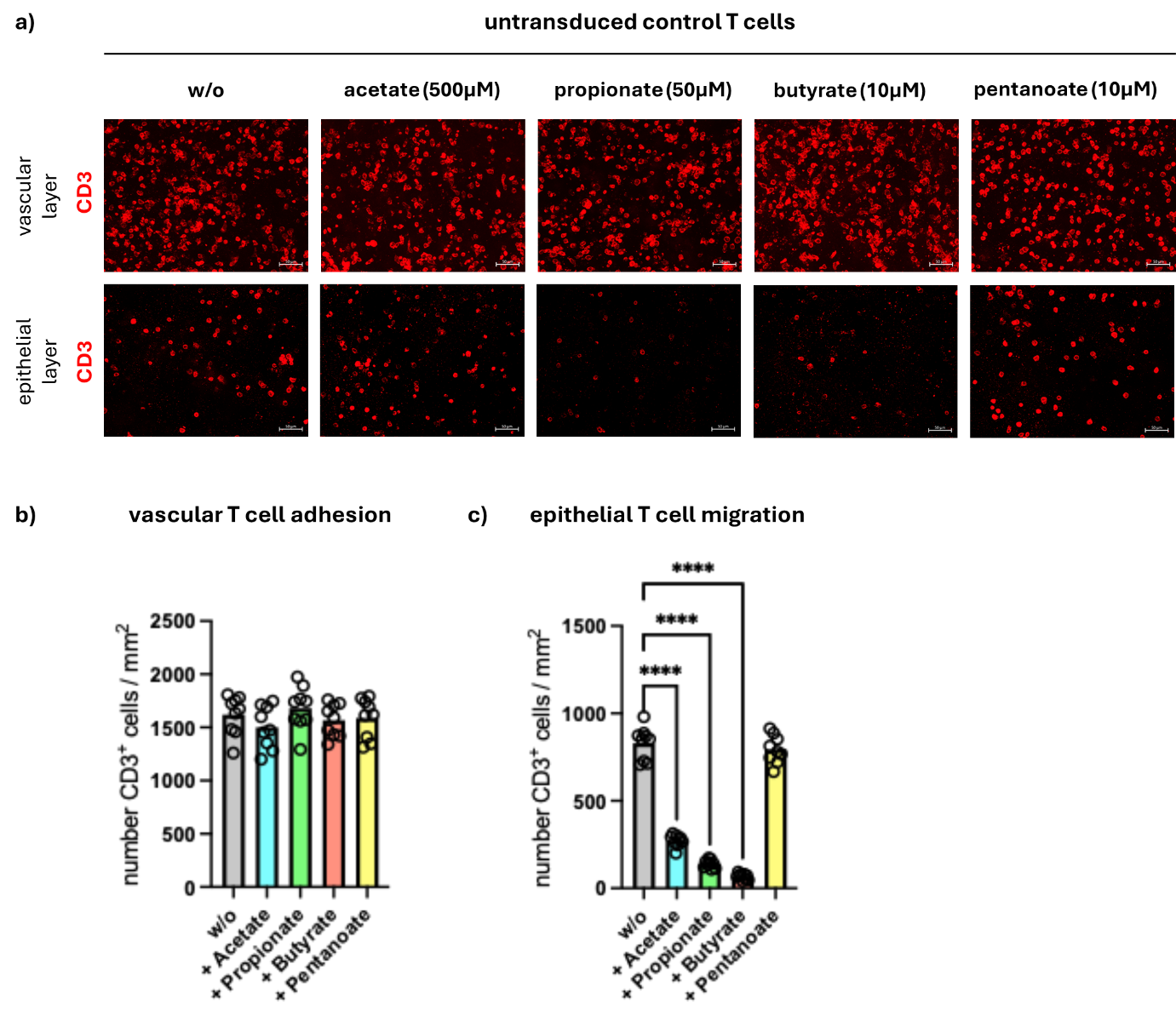


**Supplementary Figure 9. SCFA-dependent migration of untransduced control** **T-cells.** a) T-cells were stained for CD3 (red) (scale bars represent 50 µm). T-cell infiltration was quantified for b) the vascular layer and c) the epithelial layer of the IAC model. Statistical significance was determined by using a one-way ANOVA with Tukey's multiple comparison test. Bars represent mean ± SD of 3 independent experiments (n = 3) with three data points per replicate. ****p < 0.0001.


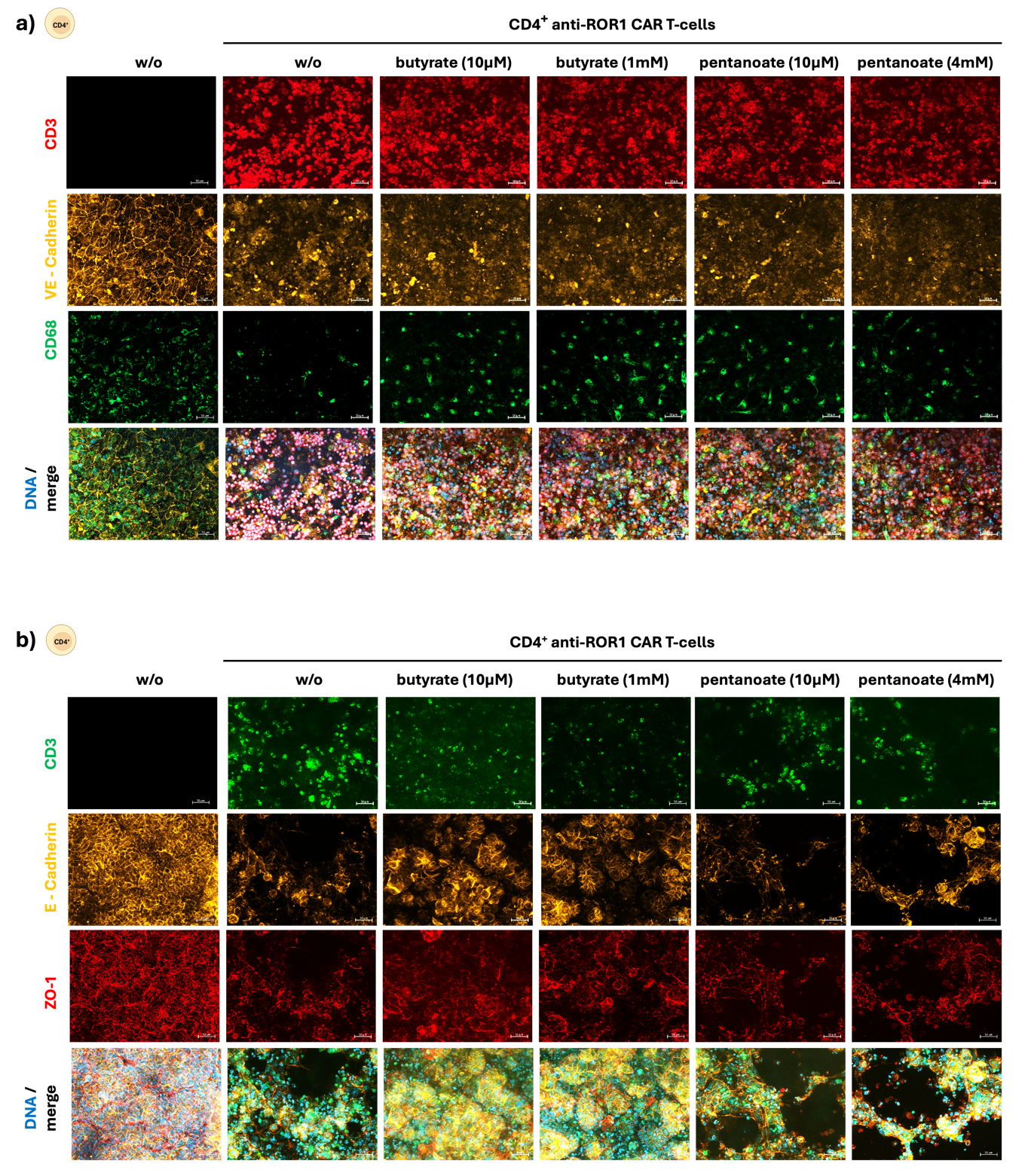


**Supplementary Figure 10. IAC model perfused with CD4^+^ anti-ROR1 CAR T-cells preincubated for 72 hours with SCFAs.** CD4^+^ anti-ROR1 CAR T-cells were preincubated for 72 hours with butyrate or pentanoate at serum-level SCFA concentrations or luminal-level SCFA concentrations before perfusion in the IAC model. a) The vascular layer was stained for CD3 (red), VE-Cadherin (yellow), CD68 (green), and merged channels including DNA (DAPI, blue) and b) the epithelial layer was stained for CD3 (green), E-Cadherin (yellow), ZO-1 (red), and merged including DNA (DAPI, blue). Scale bars represent 50 µm.

**
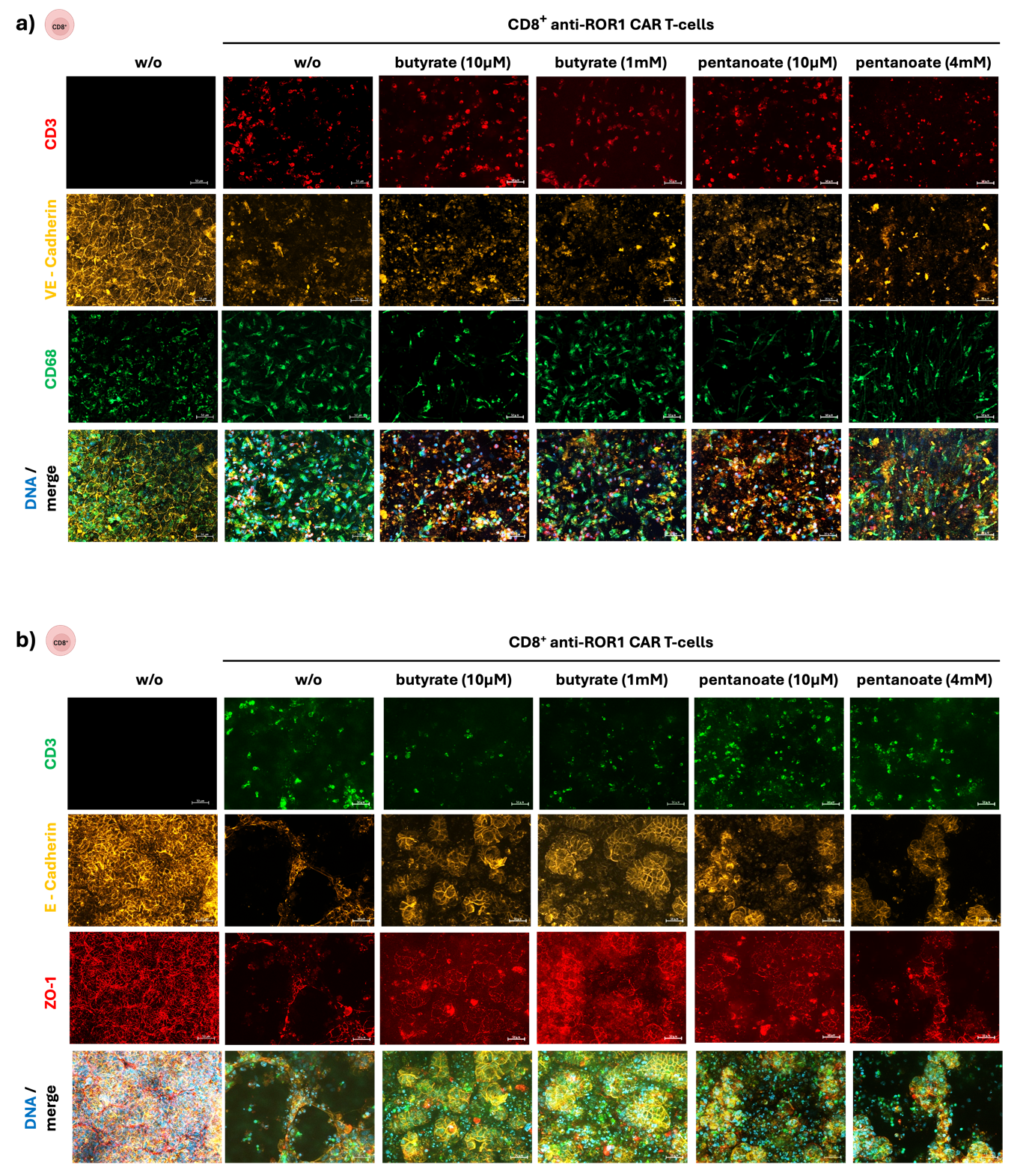
**

**Supplementary Figure 11.** **IAC model perfused with CD8^+^ anti-ROR1 CAR T-cells preincubated for 72 hours with SCFAs.** CD8^+^ anti-ROR1 CAR T-cells were preincubated for 72 hours with butyrate or pentanoate at serum-level SCFA concentrations or luminal-level SCFA concentrations before perfusion in the IAC model. a) The vascular layer was stained for CD3 (red), VE-Cadherin (yellow), CD68 (green), and merged channels including DNA (DAPI, blue) and b) the epithelial layer was stained for CD3 (green), E-Cadherin (yellow), ZO-1 (red), and merged including DNA (DAPI, blue). Scale bars represent 50 µm.


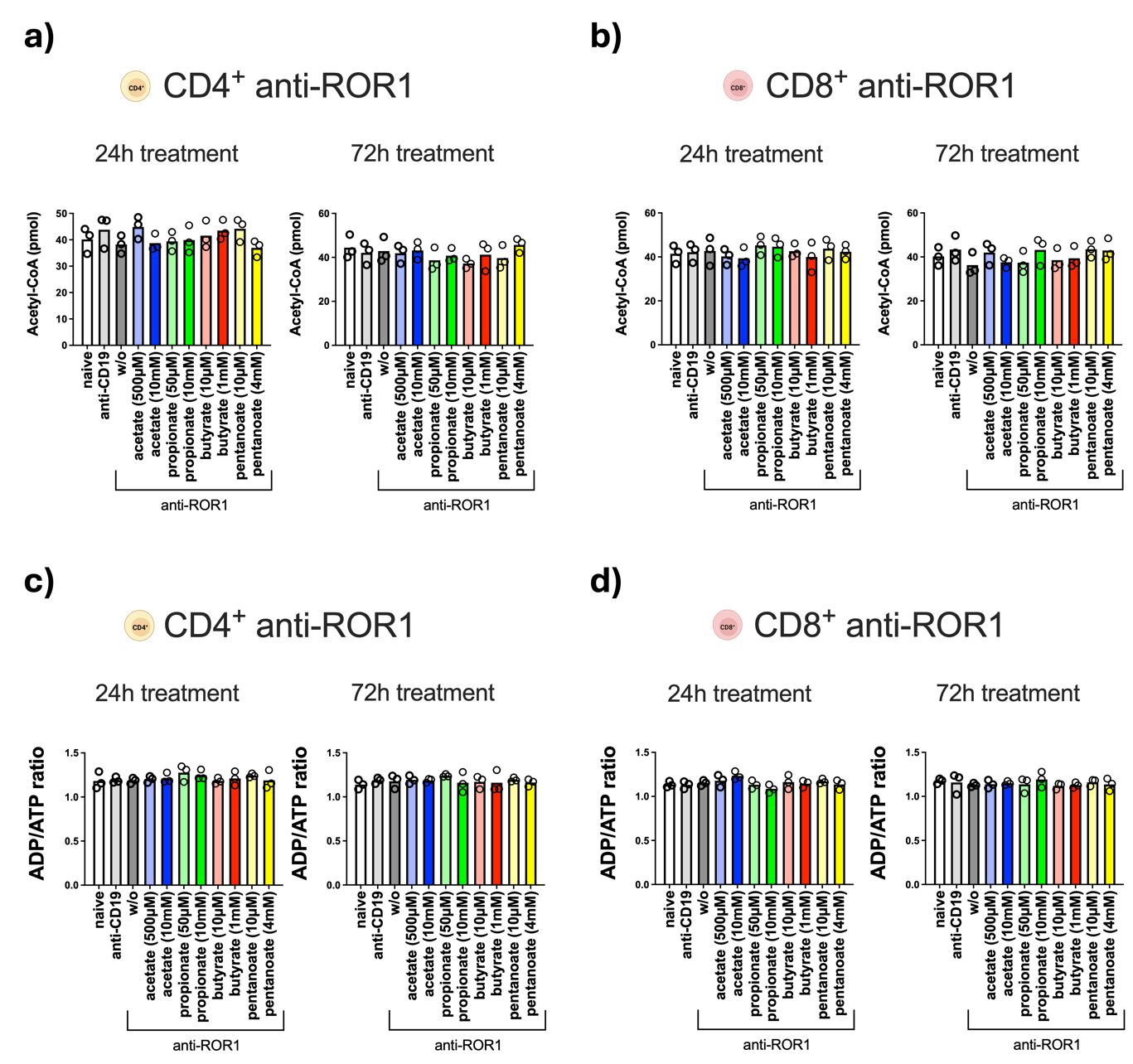


**Supplementary Figure 12. Influence of SCFA treatment on** **Acetyl-CoA and cellular ADP/ATP levels of** **CD4^+^ and CD8^+^ anti-ROR1 CAR T-cells.** a, c) CD4^+^ and b, d) CD8^+^ anti-ROR1 CAR T-cells were treated with SCFAs at serum or luminal concentration levels for 24 or 72 hours. Bars represent mean ± SD of 3 independent experiments (n = 3) with three data points per replicate.


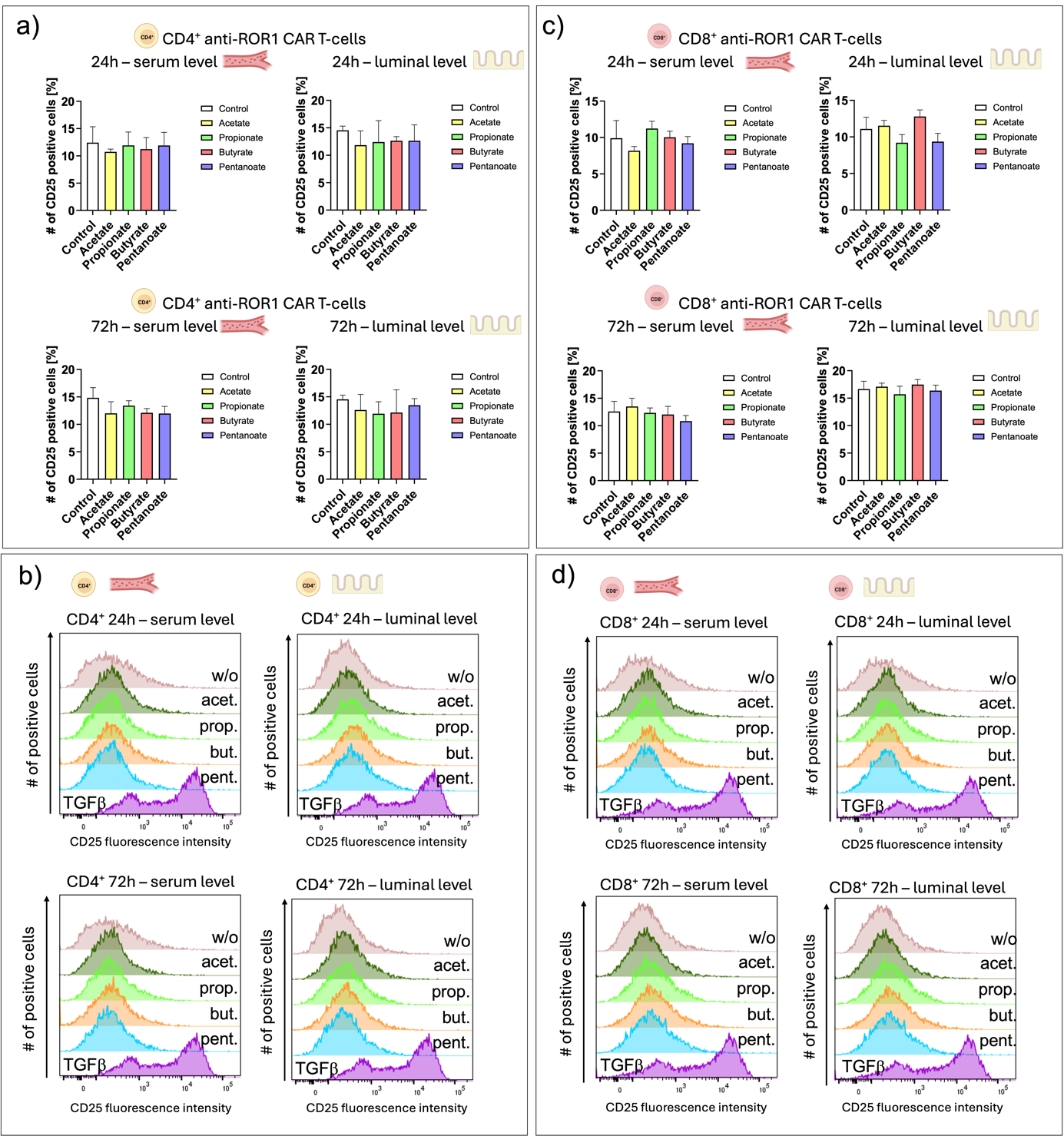


**Supplementary Figure 13. CD25 expression of CD4^+^ and CD8^+^ anti-ROR1 CAR T-cells.** CD4^+^ and CD8^+^ anti-ROR1 CAR T-cells were preincubated for 24 or 72 hours with SCFAs at serum or at luminal concentrations. Bar diagrams of three independent experiments for a-b) CD4^+^ and c-d) CD8^+^ anti-ROR1 CAR T-cells. Overlay plots show representative data from three independent experiments for b) CD4^+^ and d) CD8^+^ anti-ROR1 CAR T-cells. Treatment of CAR T-cells with 1 ng/ml TGFβ, for the indicated times, served as a positive control for CD25 upregulation. Statistical significance was determined using a one-way ANOVA with Tukey's multiple comparison test. Bars represent mean ± SD of 3 independent experiments (n = 3)


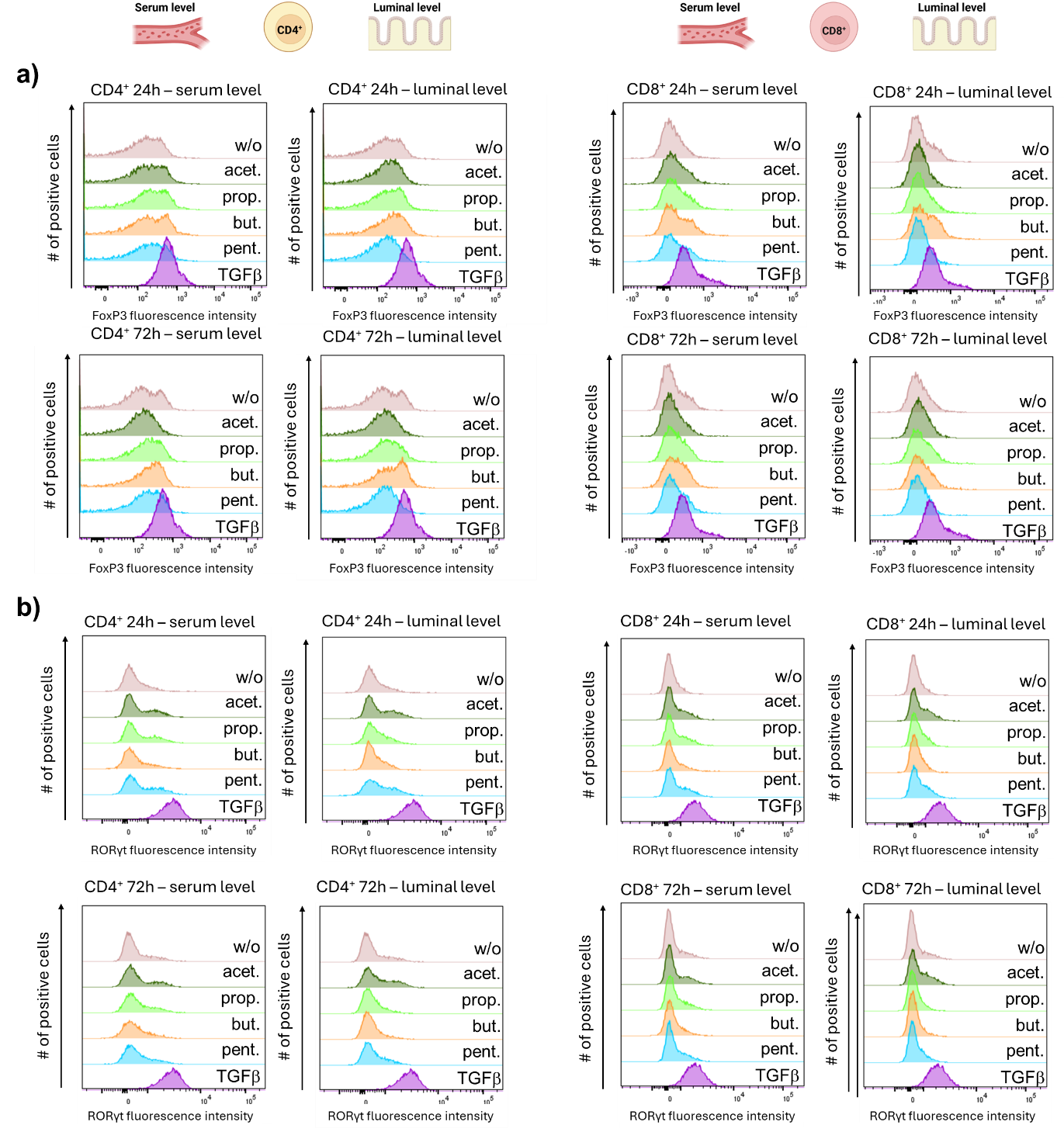


**Supplementary Figure 14. Representative overlay plots of the flow cytometric analysis of CD4^+^ and CD8^+^ anti-ROR1 CAR T-cells.** The left side shows CD4^+^ anti-ROR1 CAR T-cells and the right side CD8^+^ anti-ROR1 CAR T-cells of a) FoxP3 and b) RORγt^+^.


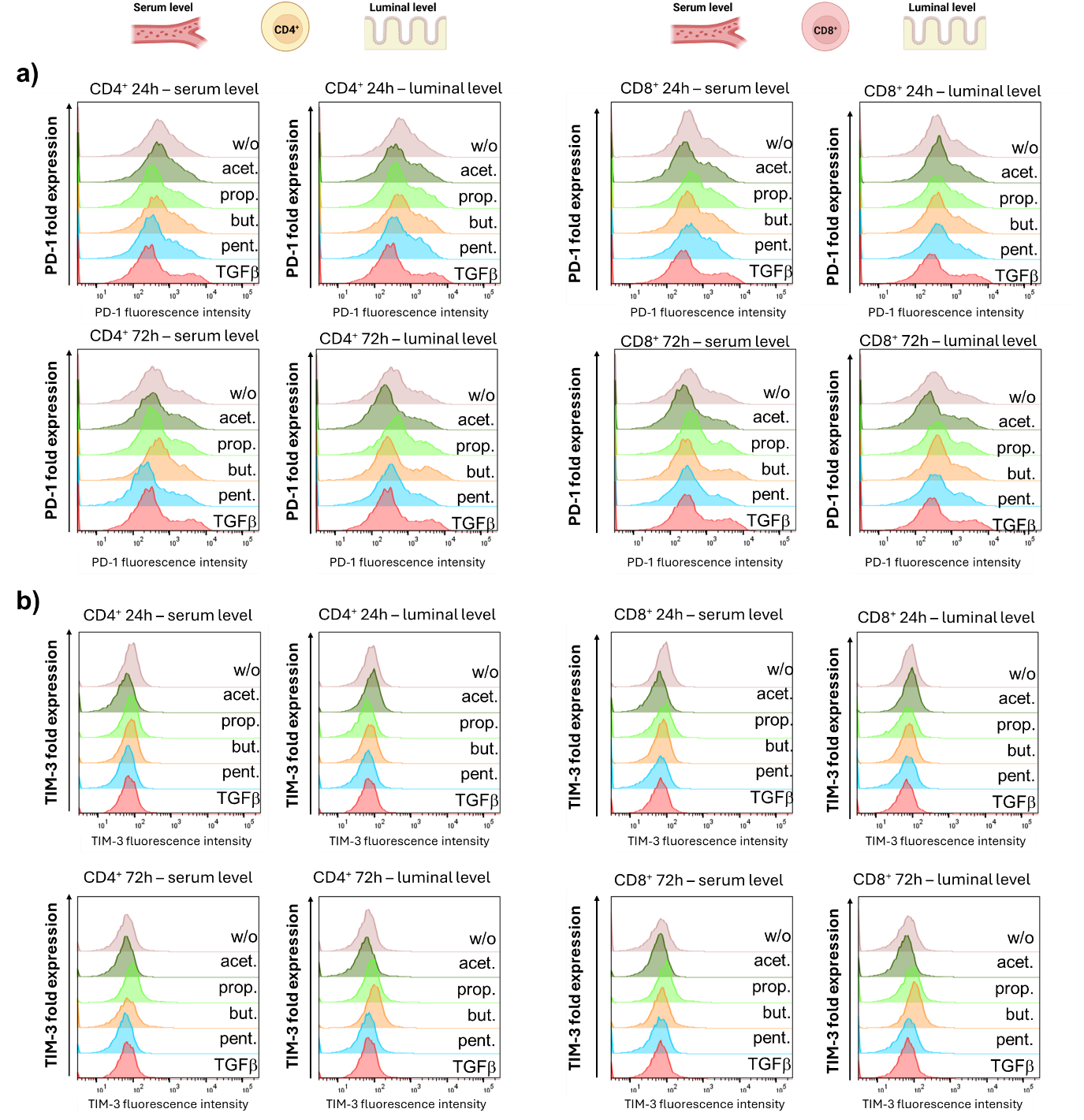


**Supplementary Figure 15. Representative overlay plots of the flow cytometric analysis of CD4^+^ and CD8^+^ anti-ROR1 CAR T-cells.** The left side shows CD4^+^ anti-ROR1 CAR T-cells and the right side CD8^+^ anti-ROR1 CAR T-cells of a) PD-1 and b) TIM-3.


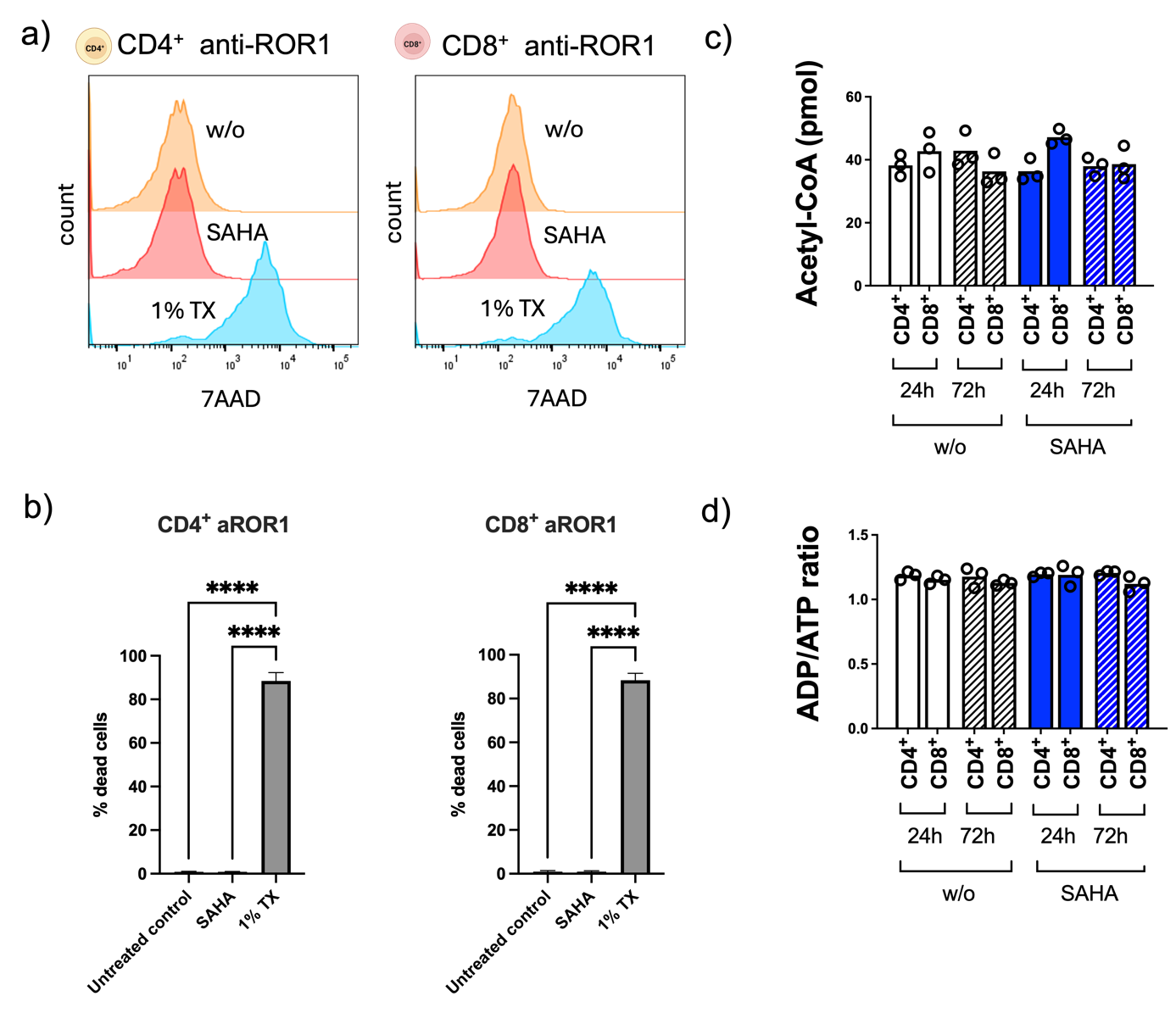


**Supplementary Figure 16.** **Effects of SAHA treatment on cell viability, acetyl-CoA, and ADP/ATP ratio within CD4^+^ and CD8^+^ anti-ROR1 CAR T-cells.** a) and b) Viability assay of CD4^+^ and CD8^+^ anti-ROR1 CAR T-cells without treatment (w/o), treated with SAHA and Triton X-100 (TX) for 72 hours, a) representative overlay plots and b) quantification of three independent experiments c) acetyl-CoA levels and d) ADP/ATP ratio of CD4^+^ and CD8^+^ anti-ROR1 CAR T-cells without treatment (w/o) or with SAHA treatment for 24 or 72 hours. Statistical significance was determined using a one-way ANOVA with Tukey's multiple comparison test. Bars represent mean ± SD of 3 independent experiments (n = 3). ****p < 0.0001.

**
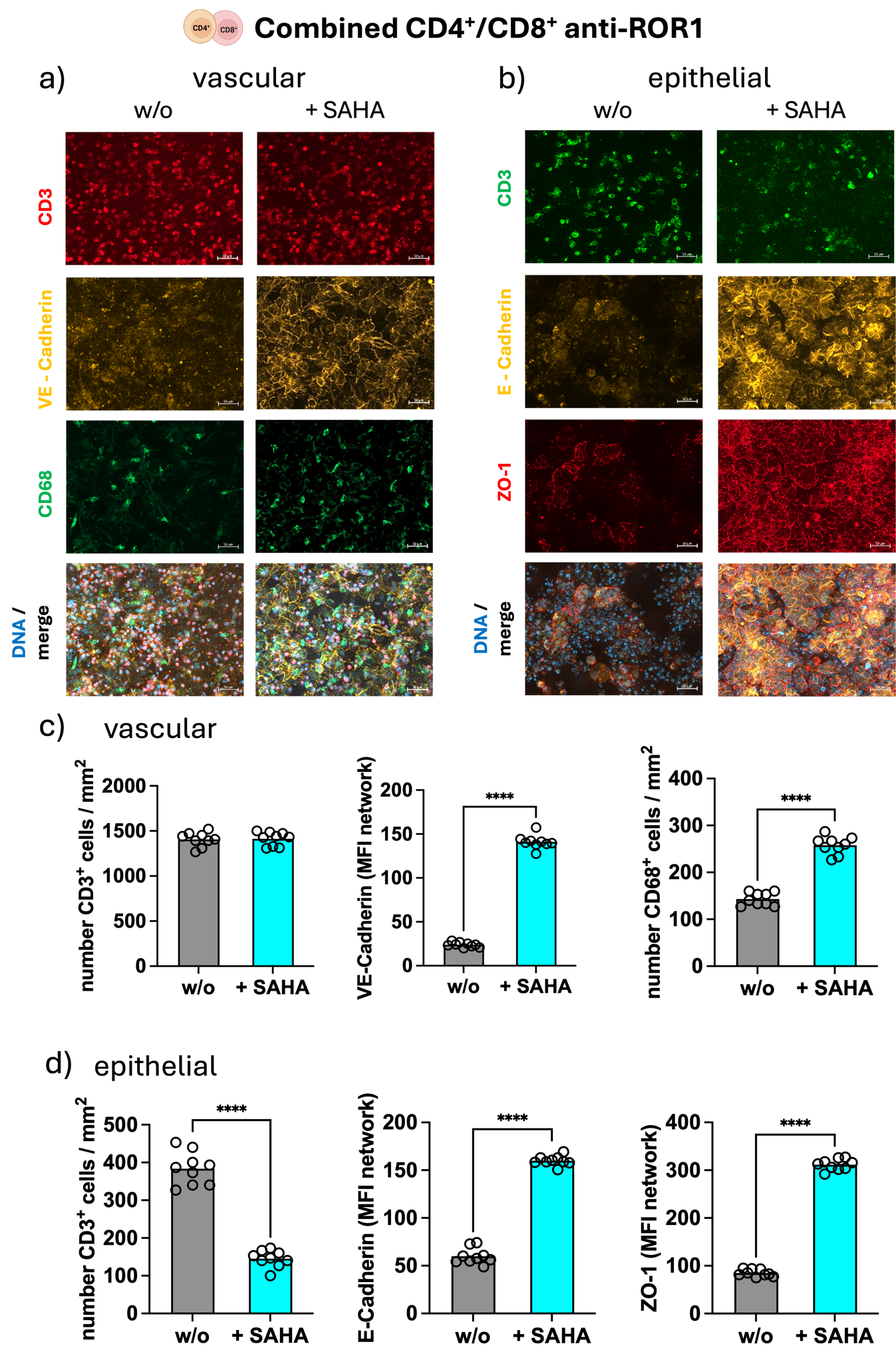
**

**Supplementary Figure 17.** **Effects of SAHA treatment (24 hours) on CAR T-cells in the IAC model.** A 1:1 mix of CD4^+^ and CD8^+^ anti-ROR1 CD4^+^ and CD8^+^ anti-ROR1 CAR T-cells was preincubated for 24 hours with SAHA and perfused in the IAC model. a) Vascular side stained for CD3 (red), VE-Cadherin (yellow), CD68 (green), and merged channels including DNA (DAPI, blue). b) Epithelial layer stained for CD3 (green), E-Cadherin (yellow), ZO-1 (red), and merged including DNA (DAPI, blue). c) and d) show the corresponding quantification. Scale bars represent 50 µm. Statistical significance was determined using a one-way ANOVA with Tukey's multiple comparison test. Bars represent mean ± SD of 3 independent experiments (n = 3) with three data points per replicate. ****p < 0.0001.
